# Regulation of Transgene Expression by the Natural Sweetener Xylose

**DOI:** 10.1002/advs.202203193

**Published:** 2022-10-31

**Authors:** Silvia Galvan, Oliver Madderson, Shuai Xue, Ana P. Teixeira, Martin Fussenegger

**Affiliations:** ^1^ Department of Biosystems Science and Engineering ETH Zurich Mattenstrasse 26 Basel CH‐4058 Switzerland; ^2^ Faculty of Life Science University of Basel Mattenstrasse 26 Basel CH‐4058 Switzerland

**Keywords:** diabetes, gene regulation, gene switches, sweeteners, synthetic biology

## Abstract

Next‐generation gene and engineered‐cell therapies benefit from incorporating synthetic gene networks that can precisely regulate the therapeutic output in response to externally administered signal inputs that are safe, readily bioavailable and pleasant to take. To enable such therapeutic control, a mammalian gene switch is designed to be responsive to the natural sweetener xylose and its functionality is assessed in mouse studies. The gene switch consists of the bacterial transcription regulator XylR fused to a mammalian transactivator, which binds to an optimized promoter in the presence of xylose, thereby allowing dose‐dependent transgene expression. The sensitivity of SWEET (sweetener‐inducible expression of transgene) is improved by coexpressing a xylose transporter. Mice implanted with encapsulated SWEET‐engineered cells show increased blood levels of cargo protein when taking xylose‐sweetened water or coffee, or highly concentrated apple extract, while they do not respond to intake of a usual amount of carrots, which contain xylose. In a proof‐of‐concept therapeutic application study, type‐1 diabetic mice engineered with insulin‐expressing SWEET show lowered glycemia and increased insulin levels when administered this fairly diabetic‐compliant sweetener, compared to untreated mice. A SWEET‐based therapy appears to have the potential to integrate seamlessly into patients’ life‐style and food habits in the move toward personalized medicine.

## Graphical abstract

Next‐generation gene and cell‐therapies require precise regulation, safety, and patient compliance. Here, a new gene switch responsive to the natural sweetener xylose is developed. This system enables precise control of desired gene expression in vivo after the consumption of xylose‐containing food or drinks. The sweetener‐inducible expression of transgene (SWEET) platform would be easily integrated into dietary routines for the treatment of a disease state.

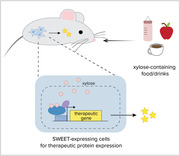

## 1 Introduction

In recent years, a few gene and engineered‐cell therapies have achieved regulatory approval for the treatment of life‐threatening diseases. Often, however, little to no control can be exerted over these therapies once they have been delivered to a patient, and this constitutes a serious limitation, especially for diseases where therapeutic windows are narrow and patient‐specific. For example, adverse effects have been reported in numerous patients receiving CAR‐T cell immunotherapies, where excessive activation of the therapeutic cells caused severe cytokine storms.^[^ ^1^, ^2^ ^]^ Precise regulation over the dosage and timing of the therapeutic program is needed to achieve improved safety and efficacy outcomes and to facilitate successful clinical translation of next‐generation gene‐ or cell‐based therapies. To address these challenges, there is a vast toolbox of trigger‐inducible synthetic gene circuits that are controlled by small‐molecular compounds,^[^ ^3^, ^4^ ^]^ such as antibiotics,^[^ ^5^ ^]^ vitamins,^[^ ^6^ ^]^ metabolites,^[^ ^7^ ^]^ plant‐derived compounds,^[^ ^8^, ^9^ ^]^ food additives,^[^ ^10^, ^11^ ^]^ or cosmetics.^[^ ^12^ ^]^ Externally modulated gene switches have been tested in preclinical studies to treat experimental diabetes,^[^ ^13^ ^]^ cancer,^[^ ^14^ ^]^ and chronic pain.^[^ ^15^ ^]^ Nevertheless, the available inducers often suffer from significant shortcomings that compromise their regulatory performance in a therapeutic setting, including poor bioavailability, pleiotropic side‐effects, unfavorable pharmacodynamics, lack of orthogonality, or poor patient compliance.^[^ ^16^ ^]^ Therefore, the quest for superior trigger‐inducible synthetic circuits combining specific, tunable, and reversible expression of therapeutic genes with appropriate dosing dynamics is still ongoing.

Xylose represents an excellent inducer candidate from a therapeutic point of view. This pentose sugar, isolated from wood, is commercially available and also naturally present in small quantities in a variety of whole fruits and vegetables,^[^ ^17^, ^18^, ^19^ ^]^ and it features on the GRAS (generally recognized as safe) list of regulators.^[^ ^20^ ^]^ Moreover, it has been routinely administered to patients as part of the xylose tolerance test to diagnose possible problems in the absorption of nutrients at the level of the small intestine, as xylose is normally easily absorbed in the intestines.^[^ ^21^, ^22^, ^23^ ^]^ Furthermore, xylose has a sweet taste while containing fewer calories than the commonly consumed table sugar. While expression of xylose‐metabolizing enzymes has not been detected in human cells, a small part of ingested xylose can be metabolized by the gut microbiota^[^ ^24^ ^]^ and the rest is then largely excreted by the kidneys. This combination of properties inspired us to engineer a mammalian gene switch triggered by this natural sweetener. *Escherichia coli* is able to utilize xylose as an alternative carbon source, enabling it to rapidly adapt to a glucose‐limited environment.^[^ ^25^ ^]^ The synthesis of proteins required for xylose metabolism in *E. coli* is regulated by the transcription factor XylR, which is responsible for the activation of xylose‐responsive genes. XylR is a 392‐amino‐acid protein with an N‐terminal xylose‐binding domain and a C‐terminal DNA‐binding domain. Xylose binds to XylR antiparallel dimers, inducing structural changes that allow XylR to bind at the DNA operator sites to promote gene expression.^[^ ^26^ ^]^ Capitalizing on the basic components involved in xylose regulation in *E. coli*, we have designed SWEET (sweetener‐inducible expression of transgene) as an orthogonal, highly sensitive and precise xylose‐responsive mammalian gene switch. First, we optimized and characterized the xylose gene switch in vitro and created a more sensitive version by combining it with a heterologous xylose transporter. Then, we confirmed the functionality of SWEET in vivo as a cell‐based or gene‐based platform. We demonstrated that SWEET could be easily integrated into the diet by consuming xylose‐sweetened beverages, such as water or coffee, or by drinking selected fruit extracts, while it is not activated by the ingestion of usual quantities of fruits and vegetables. Finally, we confirmed that DNA‐delivered SWEET_ins_ (insulin‐expressing SWEET) can alleviate hyperglycemia in type‐1 diabetic (T1D) mice by producing insulin in response to xylose administration over a prolonged period of time. Because of the sensitivity, precision, short inducer blood half‐life,^[^ ^27^ ^]^ and ease of administration, we believe therapies based on SWEET could be easily integrated into people's lifestyle and personalized to accommodate their routines, and should have a positive impact on their quality of life.

## 2 Results

### 2.1 Design of a Mammalian Xylose Gene Switch

**Figure 1:**
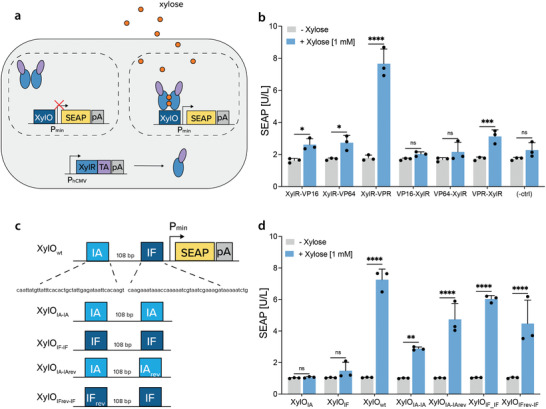
Engineering of a xylose‐inducible gene switch in mammalian cells. a) Schematic representation of the genetic components required for the engineering of a xylose gene switch in mammalian cells. In the absence of xylose, the constitutively expressed XylR fused to a mammalian transactivation (TA) domain cannot bind to the xylose operator sequence (XylO) upstream of a minimal promoter (P_min_) and therefore the expression of the downstream reporter gene (SEAP) is turned off. In the presence of xylose, the XylR‐TA dimer changes conformation and becomes XylO‐binding‐competent to activate SEAP expression. b) Screening of mammalian transactivation domains fused to the N‐ or C‐terminus of the xylose‐responsive XylR protein. HEK293T cells were transfected with XylR fused to VP16, VP64, or VPR, either at the N‐ or C‐terminus. Transfected cells with constitutive expression of one of the synthetic transcription factor variants and a reporter plasmid with a synthetic promoter bearing the native *E. coli xylO* operator sequence driving SEAP expression (XylO_wt_) were grown in standard DMEM medium or in medium supplemented with 1 mm xylose for 20 h. Cells transfected with the reporter plasmid and an empty vector to match the same total amount of DNA were included as a negative control (‐ctrl). c) Design of the promoter variants which were screened for the ability to activate SEAP expression. The top construct (pOM213) shows the wild‐type *E. coli* operator sequence, which consists of two 42 bp sites, named IA and IF, spaced by 108 bp. For mammalian gene expression, this sequence (XylO_wt_) is positioned upstream of a minimal promoter (P_min_). The remaining constructs have different combinations of the IA and IF sequences, keeping the same 108 bp spacing between them, namely XylO_IA–IA_ (pSG164), XylO_IF–IF_ (pSG165), XylO_IA–IArev_ (pSG166), XylO_IFrev–IF_ (pSG167). d) SEAP expression from HEK239T cells transfected with pOM219 (XylR‐VPR) and one of the reporter plasmids bearing the promoter regions represented in c), or only one IA (XylO_IA_, pOM211) or IF (XylO_IF_, pOM212) site. Transfected cells were grown in medium with or without 1 mm xylose for 20 h before SEAP quantification. Data are shown as mean ± SD of *n* = 3 biologically independent samples, representative of *n* = 3 independent experiments (the individual data points are shown as dots). Statistical significance was calculated by two‐way ANOVA. ns: not significant, *P < 0.05, **P < 0.01, ***P < 0.001, ****P < 0.0001.

To build a mammalian gene switch regulated by the low‐calorie sweetener xylose (**Figure** **1**a), we fused the xylose‐responsive transcriptional regulator XylR from *E. coli* to three different mammalian transactivation domains, namely the VP16 (herpes simplex virus activation domain), VP64 (four tandem repeats of VP16), and VPR (three fused domains VP64‐p65‐Rta, VP64, p65, and the Epstein‐Barr virus R transactivator). Although VPR is considered to be a stronger transactivator, for some gene switches it does not provide better performance than VP16 or VP64, either in terms of fold‐induction (VPR‐based gene switches tend to be leakier) or maximum expression levels achieved.^[^ ^28^ ^]^ Therefore, we decided to screen the three transactivators fused at the N‐ or C‐terminus of XylR, using the same small linker (Ala‐Ser), to select for the ability to provide higher fold‐induction, minimal leakiness and reporter absolute values in a xylose‐dependent manner. The transcription factors were constitutively expressed in human embryonic kidney (HEK293T) cells along with the reporter protein human placental secreted alkaline phosphatase (SEAP) controlled by a synthetic promoter containing the wild type *E. coli xylO* operator sequence (pOM213; XylO_wt_‐SEAP‐pA_bGH_). Among the six tested transcription factors, the fusion to the strong transactivator VPR at the C‐terminus of XylR (pOM219; P_hCMV_‐XylR‐VPR‐pA_bGH_) yielded the best fold‐induction of SEAP expression with comparable background activation to VP16 and VP64 when cells were cultured in the presence of xylose (Figure 1b).

Next, we set out to explore the effect of modifying the XylR‐binding promoter region. In *E. coli*, the XylR transcription factor regulates two cotranscribed operons organized in opposite directions (*xylAB* and *xylFGH*) by binding to two 42‐bp‐long sequences (named IA and IF) present in the promoter region. In order to identify which promoter sequences influence gene expression in a mammalian cell setting, we designed and screened different operator sequences for comparison with XylO_wt_: IA alone (pOM211; XylO_IA_‐SEAP‐pA_bGH_), IF alone (pOM212; XylO_IF_‐SEAP‐pA_bGH_), two IA repeats (pSG164; XylO_IA–IA_‐SEAP‐pA_bGH_), two IF repeats (pSG165; XylO_IF–IF_‐SEAP‐pA_bGH_), IA and reversed IA (pSG166; XylO_IA–IArev_‐SEAP‐pA_bGH_), reversed IF and IF (pSG167; XylO_IFrev‐IF_‐SEAP‐pA_bGH_). The design of each construct retains the wild‐type spacing of 108 bp between the two repeats (Figure 1c). Significantly increased SEAP production was observed in the presence of xylose whenever two repeats were used, regardless of the combination (Figure 1d). Nevertheless, the highest SEAP levels and fold‐induction were achieved with the wild‐type promoter, suggesting that the IA and IF sequences in the wild‐type configuration are both necessary to ensure optimal gene expression in mammalian cells.

### 2.2 Optimization of the Mammalian Xylose Gene Switch

**Figure 2:**
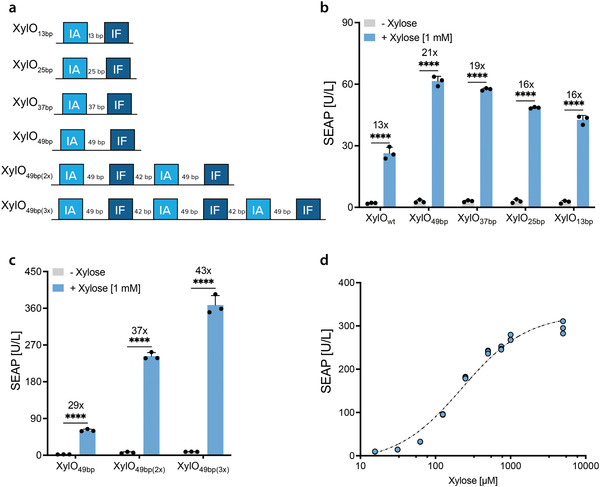
Optimization of the xylose‐inducible gene switch. a) Schematic representations of synthetic promoter regions, consisting of different spacers between IA and IF, in which the native sequence between IA and IF was reduced to a 49 (XylO_49bp_), 37 (XylO_37bp_), 25 (XylO_25bp_), or 13 (XylO_13bp_) bp sequence downstream of IA. The two bottom constructs have two or three repeats of XylO_49bp_; these were designed by adding a randomly generated 42 bp sequence between each repeat. b) Effect of truncating the spacing between IA and IF sequences in the native XylO promoter sequence. SEAP levels in the culture supernatants of HEK293T cells cotransfected with pOM219 and one of three reporter plasmids bearing the native 108 bp (XylO_wt_), or 49 bp (XylO_49bp_), 37 bp (XylO_37bp_), 25 (XylO_25bp_), or 13 (XylO_13bp_) bp spacing between the IA and IF sequences. Transfected cells were grown in medium with or without 1 mm xylose for 24 h. c) Effect of adding multiple repeats of the IA–IF sequences with optimized spacing. SEAP levels in the culture supernatants of HEK293T cells cotransfected with pOM219 and a reporter plasmid bearing one (XylO_49bp_), two (XylO_49bp(2x)_), or three (XylO_49bp(3x)_) repeats of the optimized XylR‐binding sequence. Transfected cells were grown in medium with or without 1 mm xylose for 24 h. d) Dose‐response relationship for xylose‐inducible SEAP expression. HEK293T cells cotransfected with pOM219 and pSG098 (XylO_49bp(3x)_) were treated with the indicated xylose concentrations. The calculated EC50 value was ≈224 µm. For panels b) and c) statistical data are shown as mean ± SD of *n* = 3 biologically independent samples, representative of *n* = 3 independent experiments (the individual data points are shown as dots). The significance was calculated by two‐way ANOVA. ****P < 0.0001. Numbers above the bars indicate fold difference in SEAP expression level, calculated by dividing the mean reporter expression level in the presence of xylose by the mean expression level in the absence of xylose. In panel d), single data points from *n* = 3 biologically independent samples, representative of *n* = 3 independent experiments, are shown for each xylose concentration.

To improve the fold‐induction and SEAP levels achieved with the first generation of the xylose gene switch we screened new promoter variants, keeping the IA and IF sequences but testing different spacer lengths between these two sites (**Figure** **2**a). The region downstream of IA in the wild‐type promoter was truncated from 108 to 49 bp (pOM250; XylO_49bp_‐SEAP‐pA_bGH_), 37 bp (pOM249; XylO_37bp_‐SEAP‐pA_bGH_), 25 bp (pOM248; XylO_25bp_‐SEAP‐pA_bGH_), or 13 bp (pOM247; XylO_13bp_‐SEAP‐pA_bGH_) in order to find a shorter variant with improved performance. Each truncation resulted in higher SEAP production and fold‐induction relative to XylO_wt_ (Figure 2b). Then, we selected the IA–IF sites with the best spacer (49 bp) and constructed new promoter variants consisting of two (pSG089; XylO_49bp(2_ *_x_*_)_‐SEAP‐pA_bGH_) or three (pSG098; XylO_49bp(3_ *_x_*_)_‐SEAP‐pA_bGH_) repeats, separated by 42‐bp‐long random sequences (Figure 2a). Transient transfections of HEK293T cells with each reporter construct and treatment with xylose identified three tandem repeats of the optimized binding sequence as the best‐performing xylose‐regulated gene switch, both in terms of absolute reporter expression and inducibility (Figure 2c). The set of different xylose transcription factors was screened again with the optimized reporter plasmid (pSG098), confirming that XylR‐VPR is the most promising synthetic mammalian transcription factor to promote gene expression in the presence of xylose (Figure S1, Supporting Information). HEK293T cells cotransfected with XylR‐VPR and pSG098 and exposed to increasing concentrations of xylose (from 0 to 5 mm) showed a linear dynamic range of SEAP production levels between 60 µm and 1 mm xylose with an EC50 of ≈224 µ m (Figure 2d).

### 2.3 Development and Characterization of a High‐Sensitivity Xylose Gene Switch

**Figure 3:**
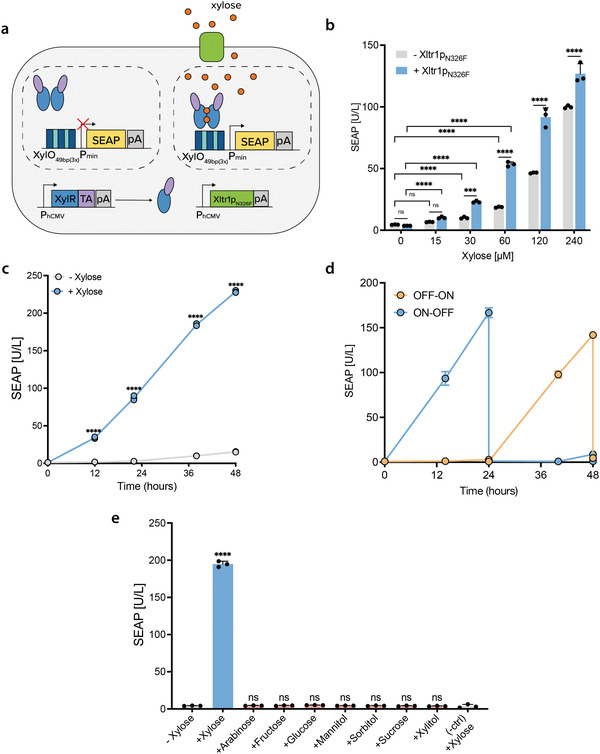
Characterization of a high‐sensitivity xylose gene switch. a) Schematic representation of the optimized genetic components that constitute SWEET. The uptake of xylose by mammalian cells is increased when they constitutively express the xylose transporter (Xltr1p_N326F_). Xylose binds to XylR‐VPR dimers which can activate reporter gene expression (SEAP) from an optimized promoter region (XylO_49bp(3x)_). b) Effect of coexpressing the heterologous xylose transporter Xltr1p_N326F_. HEK293T cells were co‐transfected with pOM219, pSG098, and either Xltr1p_N326F_ (pSG126) or an empty plasmid to maintain the total DNA concentration constant. Transfected cells were cultivated in medium containing different concentrations of xylose, from 0 to 240 µm, for 24 h. c) SWEET induction kinetics. SEAP profile of SWEET (pOM219, pSG098, pSG126)‐transfected HEK293T cells grown in medium with or without 250 µm xylose for 48 h. d) Reversibility of SWEET. HEK293T cells were transfected with SWEET and cultivated for 48 h. Medium was exchanged every 24 h, alternating between standard or xylose (250 µm)‐supplemented medium. e) SWEET specificity for xylose. SWEET‐transfected HEK293T cells were cultivated for 24 h in standard medium (−Xylose) or medium supplemented with 5 mm of one of the following sugars: xylose, arabinose, fructose, glucose, mannitol, sorbitol, sucrose, xylitol. Cells transfected with pSG098, pSG126 and an empty vector to match the same total amount of DNA were included as a negative control (‐ctrl). Data are shown as mean ± SD of *n* = 3 biologically independent samples, representative of *n* = 3 independent experiments. In panels b) and e) the individual data points are shown as dots. For panel b), statistical significance was calculated by two‐way ANOVA to compare the effect of the transporter (+Xltr1p_N326F_ vs −Xltr1p_N326F_), and to compare xylose concentrations (15, 30, 60 µm vs 0 µm). For panel c) statistical significance was calculated by two‐way ANOVA. For panel f) statistical significance was calculated by unpaired *t*‐test (vs −Xylose). ns: not significant, ***P < 0.001, ****P < 0.0001.

Xylose is not naturally metabolized by mammalian cells and its uptake may occur through unspecific sugar transporters. In order to explore whether the sensitivity of mammalian cells to low xylose concentrations could be increased, we selected heterologous xylose transporters to be coexpressed along with the transactivator XylR‐VPR and the optimized reporter construct (**Figure** **3**a). Specifically, we tested two mutant transporters, Gal2_N376F_ from *Saccharomyces cerevisiae* ^[^ ^29^ ^]^ and Xltr1p_N326F_ from *Trichoderma reesei*,^[^ ^30^ ^]^ which have been reported to show increased specificity for xylose, while abrogating the import of glucose. Although the mutant Gal2 transporter (pSG125; P_hCMV_‐Gal2_N376F_‐pA_bGH_) had no effect on SEAP gene expression (Figure S2, Supporting Information), Xltr1p_N326F_ (pSG126; P_hCMV_‐Xltr1p_N326F_‐pA_bGH_) significantly increased reporter expression in cell cultures treated with low xylose concentrations (Figure 3b), in the range of 15–250 µm. This increased sensitivity might be suitable for eventual therapeutic application. We named the optimized three‐component xylose gene switch SWEET (sweetener‐inducible expression of transgene).

Next, we characterized the expression kinetics and reversibility of cells transiently transfected with SWEET. SEAP production by cells treated with xylose significantly increased with increasing culture time, relative to cells grown in xylose‐free medium (Figure 3c). Moreover, SEAP expression could be turned OFF and ON by sequentially switching between xylose‐free and xylose‐containing medium (Figure 3d). The good reversibility performance of SWEET was also demonstrated in stably transgenic cells (Figure S3a‐b, Supporting Information), which were sequentially cultured in the presence/absence of xylose for 96 h, switching between condition every 24 h, to obtain a robust ON‐OFF‐ON‐OFF expression pattern (Figure S3c, Supporting Information).

Finally, to evaluate the specificity of SWEET for xylose, we tested if a set of different sugars and structurally similar molecules could activate reporter gene expression, including xylitol, the hydrogenated form of xylose widely utilized as a sugar substitute. We could not detect SEAP expression in the presence of any of the screened compounds other than xylose (Figure 3e), suggesting that SWEET is highly selective for xylose as an inducer. Furthermore, cells transfected with the reporter construct alone showed no activation of SEAP expression in the presence of xylose, indicating the absence of nonspecific interaction between the customized synthetic promoter region of SWEET and endogenous mammalian transcription activators.

### 2.4 Control of Transgene Expression in Mice by Xylose

**Figure 4:**
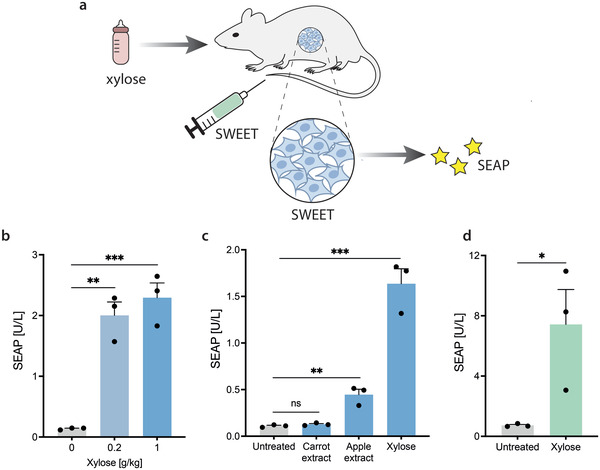
Functionality of SWEET in vivo. a) Schematic of SWEET function in vivo. Administration of xylose to the mice activates implanted encapsulated SWEET‐engineered cells, leading to SEAP expression. b) Blood SEAP levels of wild‐type mice implanted with encapsulated SWEET‐engineered cells. Mice were treated by oral gavage (o.g.) with different concentrations of xylose‐water solution. c) Performance of SWEET in mice fed with xylose‐containing fruits and vegetables. SEAP profile of mice implanted with encapsulated SWEET‐engineered cells and given either carrot extract, apple extract, or a xylose‐water solution (0.5 g kg^−1^, o.g.). d) Performance of SWEET delivered via hydrodynamic injection. Analysis of blood SEAP levels of mice injected with pSG151 and then either left untreated or treated with a xylose‐water solution (1 g kg^−1^, o.g.). Data are shown as mean ± SEM of *n* = 3 mice (the individual data points are shown as dots). Statistical significance was calculated by unpaired *t*‐test (versus untreated group of mice). *P < 0.05, **P < 0.01, ***P < 0.001.

We then investigated whether SWEET could be used to control transgene expression in vivo by implanting encapsulated SWEET‐engineered cells in mice (**Figure** **4**a). Analysis of blood samples revealed significantly increased SEAP levels in mice that received xylose by oral gavage (o.g.), regardless of the dose, relative to control mice (Figure 4b). These results suggest that xylose is absorbed in the gut, enters the systemic circulation and can stimulate implanted SWEET cells to express the regulated transgene.

The majority of people who drink coffee or tea with sugar prefer to add one or two tablespoons of a sweetener to their beverage.^[^ ^31^ ^]^ Since xylose is commonly utilized as a sweetener, we were intrigued to see if SWEET would be activated in mice given unsweetened coffee or coffee sweetened with xylose. Blood levels of SEAP were significantly higher in mice given xylose‐sweetened coffee (Figure S4, Supporting Information). To investigate whether SWEET can be regulated by dietary components, we provided mice with representative xylose‐containing fruits and vegetables: apples^[^ ^17^ ^]^ and carrots^[^ ^19^ ^]^ (Figure 4c). Mice that were fed the human equivalent of two Golden Delicious apples showed a small but significant increase in blood SEAP values. On the contrary, the extract from two medium‐sized carrots did not promote transgene expression, suggesting that selected sources of xylose‐containing food could be specifically implemented into the diet to control transgene expression. We also performed additional in vivo experiments in which SWEET was delivered as naked DNA via hydrodynamic tail vein injection (Figure 4a). This technique exploits high pressure, volume, and plasmid concentrations to facilitate the uptake of DNA by hepatocytes, with very low or no vector delivery to other organs, such as the heart, spleen, or kidneys.^[^ ^28^, ^32^, ^33^ ^]^ To increase the plasmid delivery efficiency, we first combined all SWEET components into a single vector (pSG151),^[^ ^34^ ^]^ which was successfully validated in vitro by measuring xylose‐inducible SEAP production (Figure S5, Supporting Information), and we then determined the optimal amount of plasmid to be delivered in vivo (Figure S6, Supporting Information). SWEET‐engineered mice that received xylose by o.g. showed significantly increased blood SEAP levels as compared with mice that did not receive the sweetener (Figure 4d). Collectively, these results indicate that both DNA‐based and cell‐based SWEET platforms are functional in vivo and can be sensitively regulated by exogenously supplemented xylose. To assess the safety of SWEET, we analyzed the immune responses by profiling inflammatory cytokines in vivo. No significant differences in TNF‐*α*, IL‐6, and IFN‐*γ* levels were observed between untreated and treated SWEET‐engineered mice, suggesting that SWEET‐expressing cells are not immunogenic (Figure S7, Supporting Information). As the delivery of SWEET through hydrodynamic tail vein injection allowed efficient in vivo gene expression, which resulted in higher blood SEAP levels compared to implanted SWEET‐engineered cells, we applied this delivery method in a follow‐up proof‐of‐concept therapeutic study.

### 2.5 SWEET Restores Glucose Homeostasis in T1D Mice

**Figure 5:**
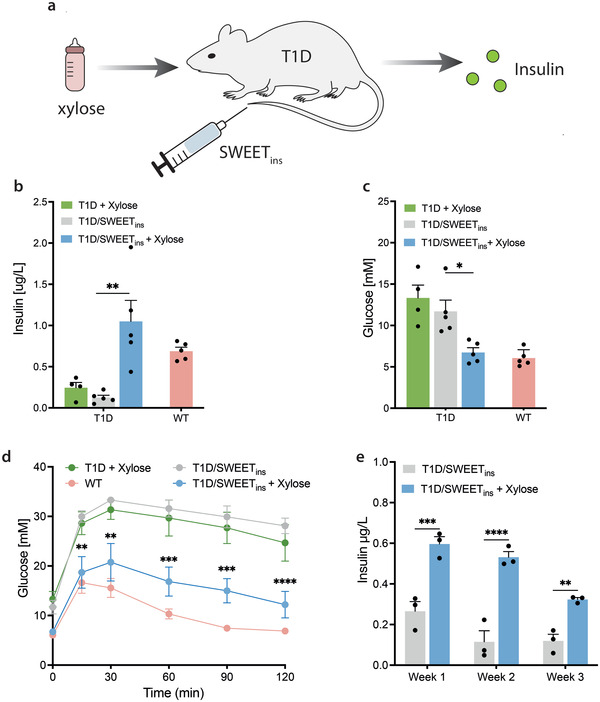
Functionality of SWEET_ins_ in vivo for the management of T1D. a) Schematic representation of T1D engineered with SWEET_ins_ via hydrodynamic injection. When xylose is administered, insulin is produced by transfected host cells. b) Fasting blood insulin levels of SWEET_ins_‐engineered T1D mice. T1D mice were injected with pSG156 and either treated (blue bar) or not (gray bar) with xylose/water solution (0.5 g kg^−1^, i.p.). Wild‐type mice (red bar) and diabetic mice treated with xylose (green bar) were included as controls. c) Fasting glucose levels of SWEET_ins_‐engineered T1D mice. Blood glucose levels of mice from the previous experiment b) were recorded. d) Glucose tolerance test (GTT) in fasting mice from previous experiments b,c). The experimental groups were: wild‐type mice (WT, red line), unengineered diabetic mice treated with xylose (T1D + Xylose, green line), SWEET_ins_‐engineered diabetic mice treated with xylose (T1D/SWEET_ins_+ Xylose, blue line), SWEET_ins_‐engineered diabetic mice that did not receive xylose treatment (T1D/SWEET_ins_, gray line). e) Fasting blood insulin profile of SWEET_ins_‐engineered T1D mice treated with drinking water sweetened with xylose. The treated group of diabetic mice (T1D/SWEET_ins_+ Xylose) was given water sweetened with xylose (5% w/v) ad libitum. Data in b–d) are shown as the mean ± SEM of *n* = 5 mice for T1D/SWEET_ins_ groups and wild‐type and *n* = 4 for T1D + xylose control group. In panel b,c) the statistical significance of differences was calculated by unpaired *t*‐test. In panel d) the statistical significance of differences between T1D/SWEET_ins_ and T1D/SWEET_ins_ + xylose was calculated by two‐way ANOVA. Data in e) are shown as the mean ± SEM of *n* = 3 mice, statistical significance was calculated by two‐way ANOVA. The individual data points in b,c,e) are shown as dots. *P < 0.05, **P < 0.01, ***P < 0.001, ****P < 0.0001 (vs Untreated).

To showcase the applicability of SWEET to regulate gene‐ or cell‐based therapies, we focused on the treatment of experimental T1D (**Figure** **5**a) as an example of a lifelong metabolic disorder, whose patients would benefit from such advanced therapies. We first placed an insulin construct under the xylose‐responsive promoter and created a single plasmid with SWEET_ins_ (pSG156), which was validated in transient transfection cell cultures for the production of insulin in the presence of xylose (Figure S8, Supporting Information). T1D mice were hydrodynamically transfected with SWEET_ins_ and either received or did not receive xylose treatment. Treated mice showed higher blood insulin levels (Figure 5b) and lower fasting glucose levels compared to the untreated group control (Figure 5c), suggesting that sufficient insulin is produced by SWEET_ins_ to regulate blood glucose levels. Moreover, xylose treatment of SWEET_ins_‐expressing T1D mice showed decreased glycemia during a glucose tolerance test (GTT) compared to the untreated mice which remained hyperglycemic (Figure 5d). We confirmed the prolonged effect of SWEET_ins_ by monitoring fasting blood glucose and insulin levels in diabetic mice until the third week after injection (Figure S9, Supporting Information). Finally, we tested the delivery of xylose in drinking water and observed that T1D mice with ad libitum access to sweetened water had significantly higher blood insulin levels than mice drinking xylose‐free water (Figure 5e). Collectively, these results indicate that SWEET_ins_ is a robust system and can safely control hyperglycemia in T1D mice. Moreover, the delivery of SWEET_ins_ via hydrodynamic injection was able to manage the disease for 3 weeks, suggesting that a DNA‐based delivery method would be suitable for a longer‐term therapy.

## 3 Discussion

For future engineered‐cell or gene‐based therapeutic strategies, we need to consider the selection of optimal triggers to enable the delivery of therapeutic proteins at effective doses, while maintaining high controllability of the system to maximize safety. The exogenous stimulus should be carefully selected to ensure little or no toxicity or side effects, tight regulation of the transgene, and short half‐life in vivo, and should be FDA‐approved or clinically licensed. In most cases, advanced therapies targeting chronic diseases need to operate over prolonged periods of time, and therefore the external trigger has to be easily administered, inexpensive, and compatible with long‐term application in humans. Antibiotics,^[^ ^5^ ^]^ drugs,^[^ ^35^, ^36^ ^]^ vitamins,^[^ ^6^ ^]^ and hormones^[^ ^37^ ^]^ generally do not meet these requirements, given their broad spectrum of toxicity, especially when continuously administered. In contrast, because of their safety and ease of administration, food components or food additives, such as caffeine,^[^ ^9^ ^]^ menthol,^[^ ^8^ ^]^ and vanillic acid^[^ ^10^ ^]^ are emerging as promising inducers, provided that they trigger the gene switches at concentrations well above those produced by the patients’ normal dietary intake.

People suffering from type‐1 diabetes mellitus have their insulin‐producing beta cells destroyed by their own immune system leading to chronic hyperglycemia.^[^ ^38^ ^]^ These patients are commonly treated daily with multiple injections of insulin, and their blood glucose levels have to be constantly monitored, considerably affecting their quality of life.^[^ ^39^ ^]^ For this reason, the consumption of synthetic sugar substitutes, such as aspartame and saccharin, has been on the rise due to their sweet taste, null glycemic index, and usefulness for weight management and glycemic control. However, their safety and toxicity are still under debate, especially when consumed in high doses and added as sweeteners in refined foods.^[^ ^40^, ^41^, ^42^ ^]^ Xylitol, the sweeter hydrogenated form of xylose,^[^ ^43^ ^]^ would not be a good candidate as an inducer for precision therapy due to its widespread use as a food additive in commercial products, which would make it difficult to avoid accidental activation of the therapeutic output. Xylose offers multiple advantages over other sweeteners as an inducer for a T1D therapeutic strategy: it is natural, cheap, has a short blood half‐life and is absorbed independently of the insulin response. Therefore, it is safer than artificial sweeteners and is compatible with diabetic patients’ requirements. Furthermore, supplementing xylose in the human diet seems to have beneficial effects on lipid metabolism and weight management.^[^ ^44^, ^45^ ^]^ In line with xylose low price, significant efforts have been made in the field of microbial engineering to confer xylose assimilation capability upon yeast strains, such as *S. cerevisiae*, in order to obtain sustainable, large‐scale bioethanol production from lignocellulose.^[^ ^46^, ^47^, ^48^, ^49^ ^]^

In this work, we have designed and optimized a precise, robust xylose mammalian gene switch characterized by dose‐dependent transgene expression and reversible regulation. We showed that SWEET is highly specific to xylose, being insensitive to other sugars and structurally similar molecules, including xylitol. We also demonstrated the potential of insulin‐expressing SWEET (SWEET_ins_) as a candidate next‐generation therapy for the treatment of T1D, one of the most dynamically complex medical conditions. The delivery of xylose to SWEET_ins_‐equipped diabetic mice could control postprandial blood‐glucose spikes, and could also decrease glycemia and increase insulin levels over a prolonged period of time. Importantly, transgene expression was activated when animals were given a xylose‐sweetened water (corresponding to the human equivalent of a teaspoon of xylose dissolved in a solution^[^ ^50^ ^]^), coffee or concentrated apple extract. In this work, we have shown that the SWEET platform provides some key advantages, including the amenable characteristics of the inducer, as well as high functionality in vivo when delivered either as encapsulated engineered cells or as naked DNA. These properties make SWEET a flexible platform that we anticipate could be implemented for the treatment of other chronic diseases, not only T1D, in the future. Bearing in mind that a successful therapy should have a positive impact on the patient's quality of life, these findings suggest that SWEET could be easily integrated into dietary routines and life‐styles, for example, by introducing xylose‐sweetened drinks or fresh fruit extracts.

## 4 Experimental Section

### Plasmid Construction

Design and cloning details for all genetic components utilized in this study are provided in Tables S1–S3 (Supporting Information). Plasmids were designed using Benchling (www.benchling.com) and constructed by standard molecular cloning techniques using restriction digestion and ligation, Gibson assembly or mutagenesis PCR (polymerase chain reaction). For restriction enzyme‐based cloning, plasmids were digested with the desired endonucleases (New England Biolabs); digested backbones were dephosphorylated with Quick CIP (M0525L, New England Biolabs) before ligation with T4 DNA ligase (EL0011, Thermo Fisher). For Gibson assembly, the PCR products were amplified using primers that had ≈20 bp complementary sequences at each end, followed by DpnI (R0176L, New England Biolabs) digestion. Cloning was performed utilizing a custom mix of enzymes. To perform mutagenesis PCR, primers of variable length were designed to have the desired mutation/new sequence between a sequence complementary to the backbone (≈20 bp) and a sequence complementary to the other primer (≈10 bp). Whole plasmid PCR amplification products were also DpnI‐digested before being used to transform competent *E. coli* strains. All PCR reactions were performed using Q5 High‐Fidelity DNA polymerase (M0491L, New England Biolabs). After ligation, the plasmids were amplified in One shot TOP10 *E. coli* strain (C404010, Thermo Fisher) and DNA was extracted using a plasmid miniprep kit (SL‐11003321, ExtrAXON Plasmid Mini Kit) or ZymoPURE II Plasmid Midiprep Kit (D4200, Zymo Research). Constructs were verified by Sanger sequencing performed by an external vendor (Microsynth AG). Synthetic gene fragments used in the study were codon‐optimized for expression in human cells and synthesized commercially (Twist Bioscience).

### Cell Culture

Human embryonic kidney cells (HEK293T, ATCC: CRL‐11268) were cultivated in Dulbecco's modified Eagle's medium (DMEM; 10 566 016, Thermo Fischer) supplemented with 10% v/v fetal bovine serum (FBS; F7524, Sigma‐Aldrich) under a humidified atmosphere containing 5% CO_2_ at 37 °C. Passaging of preconfluent HEK293T cultures was done by detaching cells through incubation in 0.05% trypsin‐EDTA (25 300 054, Life Technologies) for 5 min at room temperature. Cells were transferred to 10 mL culture medium, centrifuged for 2 min at 280 × g, resuspended in fresh medium and reseeded at the desired cell density. Cell number and viability were quantified using a CellDrop automated cell counter (DeNovix).

### Transient Transfection

For transfection experiments in 96‐well plates (3599, Corning), cells were seeded at 1.5 × 10^4^ cells per well, 24 h before transfection. The transfection mixture for one well of 96‐well plates consisted of 50 µL of FBS‐free DMEM containing a 1:4 DNA:PEI (polyethylenimine, MW 40 000; 24 765, Polysciences) mixture, with a total DNA amount of 150 ng. After 20 min incubation at room temperature, the transfection mixture was added dropwise to the cells, which were then incubated overnight. The next morning, the culture medium was replaced with fresh FBS containing DMEM with or without xylose (X3877, Sigma‐Aldrich) and cell supernatant samples were collected 24 h later for SEAP (human placental secreted alkaline phosphatase) reporter analysis, unless otherwise indicated. For other cell culture plate formats, the protocol was adjusted as necessary taking into account the area of the plates. Additional details for transient transfections are reported in Table S4 (Supporting Information).

### Generation of Monoclonal Stable Cell Lines

Polyclonal cell lines were generated by cotransfecting HEK293T cells with a hyperactive Sleeping Beauty transposase (pTS395) expression vector in a 1:10 (w/w) ratio with pSG149, containing SB recognition sites and encoding a puromycin resistance marker and YFP fluorophore. The medium was exchanged 12 h after transfection and cells were incubated for 48 h before the addition of selection medium containing 3 µg mL^−1^ puromycin. After three passages, the polyclonal population was sorted into single cells by FACS (fluorescence‐activated cell sorting) in a 96‐well plate format and expanded to obtain monoclonal cell lines.

### SEAP Quantification

Heat‐inactivated (30 min, 65 °C) cell culture supernatants (20 µL) were transferred into a 96‐well plate (260 836, Thermo Fisher) and mixed with 80 µL water, 80 µL 2x SEAP buffer (20 mm homoarginine, 1 mm MgCl_2_, 21% v/v diethanolamine, pH 9.8), and 20 µL substrate solution containing 20 mm para‐nitrophenyl phosphate (pNPP; 128 860 100, Acros Organics BVBA). The absorbance of samples was measured at 405 nm using a Tecan Infinite M1000 plate reader (Tecan AG) at 37 °C. SEAP concentrations in serum were quantified using a chemiluminescence‐based assay (ab133077, Abcam). Briefly, 50 µL of SEAP substrate was added to 10 µL of heat‐inactivated (30 min, 65 °C) serum and incubated at room temperature for 5 min. Chemiluminescence was measured using a Tecan M1000 plate reader (Tecan AG) and concentrations were calculated from a standard curve.

### NanoLuc Quantification

Secreted NanoLuc in cell culture supernatants was measured with the Nano‐Glo Luciferase Assay System (N1110, Promega). In brief, 7.5 µL from each sample was mixed with 7.5 µL buffer/substrate mix (50:1) in 384‐well plates (781 076, Greiner Bio One) and incubated at room temperature for 10 min. Luminescence was measured with a Tecan M1000 plate reader.

### Insulin Quantification

Recombinant mINS levels in serum were quantified using a mouse insulin ELISA kit according to the manufacturer's instructions (10‐1247‐01, Mercordia). Optical density was measured at 450 nm on a Tecan M1000 plate reader and the corresponding concentrations were calculated in Prism 9 (GraphPad Software Inc) using a cubic‐spline regression based on the measured absorbances of manufacturer‐provided standard solutions.

### Animal Experiments

Eight‐week‐old male C57BL/6JRI mice (Janvier Labs Saint‐Berthevin, France) weighing ≈20–25 g was used. Animals were housed in a controlled room set at 22 °C, 50% humidity, 12‐h light‐dark cycle with ad‐libitum access to standard diet and drinking water. Animals were randomly assigned to experimental groups. Cell implants: SWEET‐transfected HEK293T cells were encapsulated into coherent alginate‐poly‐(*L*‐lysine)‐alginate beads (400 µm; 200 cells per capsule) using an Inotech Encapsulator Research Unit IE‐50R (Buechi Labortechnik AG) with the following parameters: 20 mL syringe operated at a flow rate of 400 units, 200 µm nozzle with a vibration frequency of 1200 Hz, and bead dispersion voltage of 1.4 kV. Mice were intraperitoneally injected with 1 mL of MOPS containing 5 × 10^6^ cells. Xylose solution was supplemented by oral gavage (300 µL). Blood samples were collected the day after and serum was isolated for analysis using BD Microtainer SST tubes according to the manufacturer's instructions (30 min incubation in the dark, centrifugation for 5 min at 8000×g; 365 967, Becton Dickinson).

### Hydrodynamic Plasmid Delivery

Hydrodynamic plasmid delivery: Mice were injected with 2 mL of saline containing plasmid coding for SWEET or SWEET_ins_ (4 mg kg^−1^ of DNA per mouse, unless stated otherwise) 24 h before starting inducer treatment. For the type‐1 diabetes study, C57BL/6JRI mice (male, 8–9 weeks, Janvier) were treated with 75 mg kg^−1^ per mouse per day of streptozocin (STZ; S0130, Sigma‐Aldrich) for 4 days to deplete the insulin‐producing beta cells. Fasting blood glucose was measured after 8 h of food restriction using a clinically licensed glucometer (Accu‐Check Instant, Roche). Glucose tolerance test (GTT) was performed by i.p. injection of 1.5 g kg^−1^ glucose, and glycemia was profiled at intervals for the following 2 h. For the treatment of T1D mice, xylose administration was either performed twice per day by i.p. injection (0.5 g kg^−1^), or by o.g. (0.5 g kg^−1^), or supplemented in the drinking water for 10 h (5% w/v) before subjecting the animals to fasting for 6 h, once per week for three consecutive weeks. The delivery method is specified in each figure legend.

### Cytokine Analysis

IL‐6, TNF‐*α*, and IFN‐*γ* concentrations in mouse serum were measured using a high‐sensitivity IL‐6 mouse ELISA kit (BMS603HS, ThermoFisher), a high‐sensitivity TNF‐*α* mouse ELISA kit (BMS607HS, ThermoFisher), and an IFN‐*γ* mouse ELISA kit (BMS606‐2, ThermoFisher).

### Exposure to Inducers

Xylose was dissolved in ddH_2_O to prepare 2%, 5%, or 10% w/v solutions. In Figure S3 (Supporting Information), 5% w/v xylose‐coffee solution was prepared by dissolving xylose in coffee (Lavazza Qualità Rossa, home‐brewed) 1:10 diluted with water. In Figure 4c, carrot (medium size, orange carrot) and apple (Golden Delicious, Melinda) extracts were prepared from fresh products; the amounts were calculated based on standard human and mouse weights of 60 kg and 20 g, respectively.^[^ ^50^ ^]^ In all animal experiments, treatment consisted of the delivery of two doses of the inducer with an 8 h interval the day before analysis. The method of delivery of the inducer is specified in the figure legends. All experiments involving animals were performed in accordance with the Swiss animal welfare legislation, approved by the veterinary office of the Canton Basel‐Stadt (approval no. 2879/31 996) and carried out by Shuai Xue (no. LTK4899) and according to the directives of the European Community Council (2010/63/EU), approved by the French Republic (project no. DR2018‐40v5 and APAFIS #16 753), and carried out by Shuai Xue and Ghislaine Charpin‐El Hamri (no. 69 266 309) at the University of Lyon, Institut Universitaire de Technologie (IUT), F69622 Villeurbanne Cedex, France.

### Statistical Analysis

Statistical evaluation was conducted by using unpaired Student's two‐tailed *t*‐test for comparing two sets of data and two‐way ANOVA for multiple comparisons as implemented in Prism GraphPad 9 (GraphPad Software Inc., San Diego, CA).

## Conflict of Interest

The authors declare no conflict of interest.

## Supporting information

Supporting Information

## Data Availability

The data that support the findings of this study are available from the corresponding author upon reasonable request.
