# Supplementary material for: Regulation of Transgene Expression by the Natural Sweetener Xylose
Source: Adv Sci (Weinh). 2022 Oct 31;9(34):2203193. doi: 10.1002/advs.202203193 (PMC9731693; doi:10.1002/advs.202203193)
Supplement: Supplementary file 1 — Supporting Information [file ADVS-9-2203193-s001.pdf]

ADVANCED SCIENCE
Open Access

Supporting Information

for *Adv. Sci.*, DOI 10.1002/advs.202203193

Regulation of Transgene Expression by the Natural Sweetener Xylose

*Silvia Galvan, Oliver Madderson, Shuai Xue, Ana P. Teixeira and Martin Fussenegger**

Supporting Information

Regulation of transgene expression by the natural sweetener xylose

Silvia Galvan, Oliver Madderson, Shuai Xue, Ana P. Teixeira, Martin Fussenegger[*]

S. Galvan, O. Madderson, S. Xue, A. P. Teixeira, M. Fussenegger
ETH Zurich, Department of Biosystems Science and Engineering, Mattenstrasse 26, CH-4058 Basel, Switzerland.
Email: fussenegger@bsse.ethz.ch

M. Fussenegger
University of Basel, Faculty of Life Science, Basel, Switzerland.

Contents

1. **Supporting Figures S1-S9**
2. **Supporting Tables S1-S4**

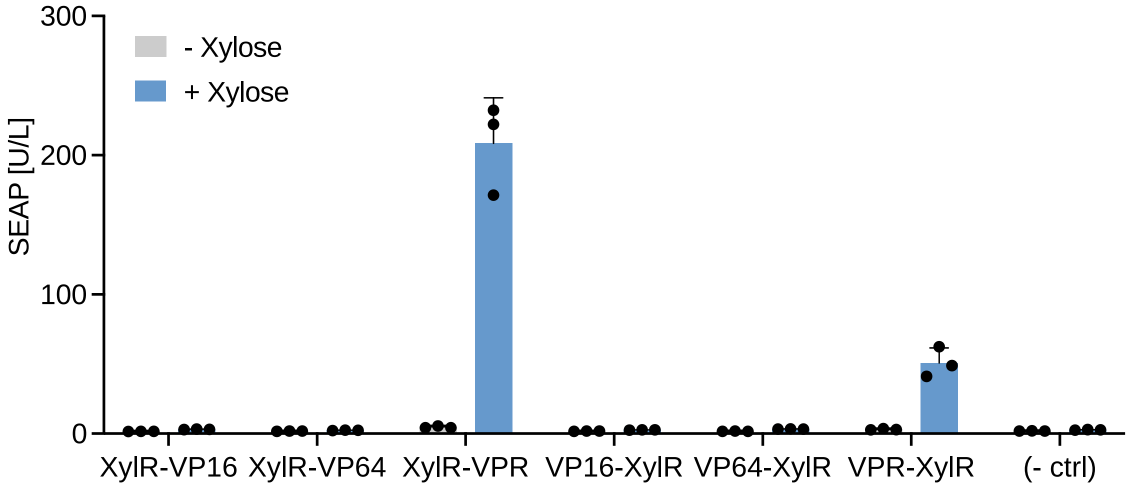

Figure S1. Screening of mammalian transactivation domains fused to the N- or C-terminus of the xylose-responsive XylR transcription factor with the optimized reporter plasmid pSG098. HEK293T cells were transfected with pSG098 and one variant of XylR fused to VP16, VP64 or VPR, either at the N- or C-terminus. Cells were cultivated in medium without or with xylose (250 µM) for 24 hours. Cells transfected with pSG098 and a mock plasmid to match the same total amount of DNA were included as a negative control (-ctrl). Data are shown as mean ± SD of n = 3 biologically independent samples, representative of n = 3 independent experiments (the individual data points are shown as dots).

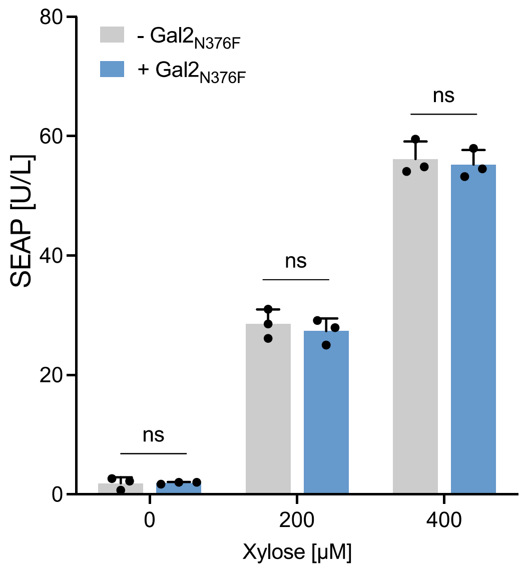

Figure S2. Co-expression of the heterologous xylose transporter Gal2$_{N376F}$ of *S. cerevisiae*. HEK293T cells were co-transfected with pOM219, pSG098 and with either pSG125 (Gal2$_{N376F}$; blue bar) or a mock plasmid (grey bar) and grown in the presence of xylose (0, 200, 400 µM). Data are shown as mean ± SD of n = 3 biologically independent samples, representative of n = 3 independent experiments (the individual data points are shown as dots). Statistical significance was calculated by two-way ANOVA. ns: not significant.

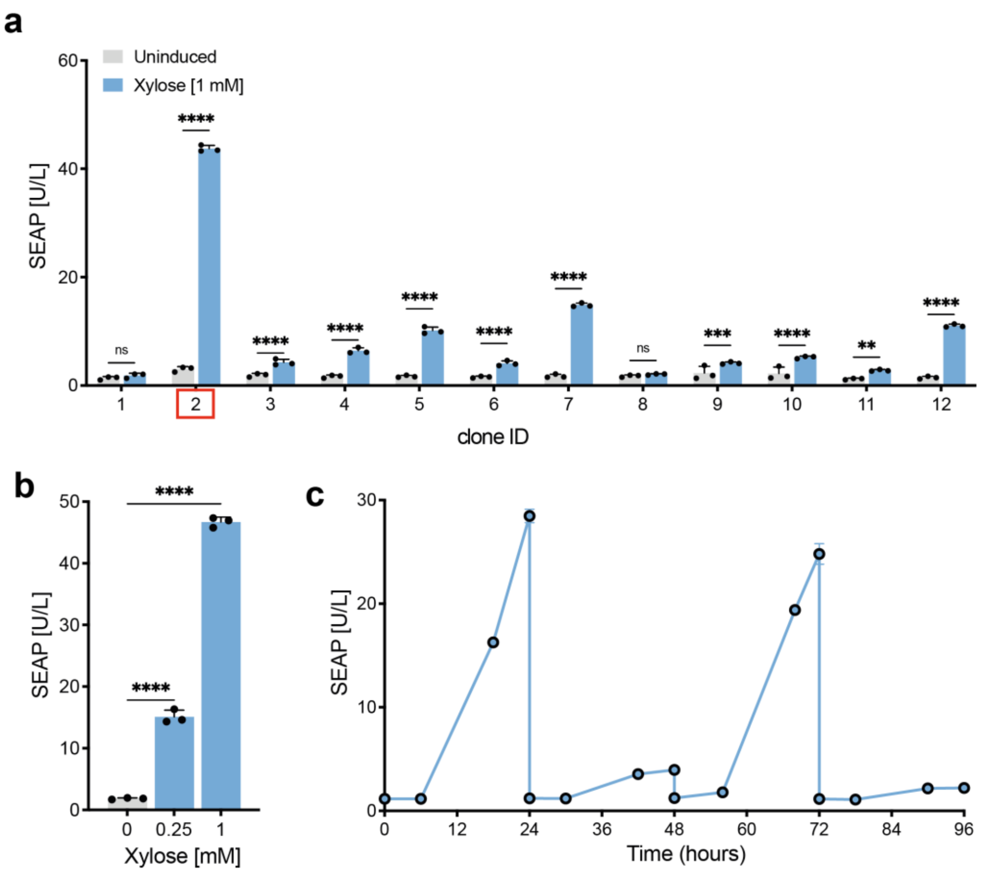

Figure S3. HEK cell line with stable genomic integration of SWEET. **a** Screening of xylose-responsive cell clones. Monoclonal cell lines derived from a cell population stably expressing SWEET (pSG149) were cultured in the absence or presence of 1 mM xylose for 24 hours before profiling SEAP production. **b** Dose-dependent SEAP production by clone #2 from **panel a** cultured in the presence of different xylose concentrations for 24 hours. **c** Reversibility of SWEET-engineered monoclonal stable cell line. The medium was exchanged every 24 hours, alternating between standard or xylose (1 mM)-supplemented medium, for a total of four days following a ON-OFF-ON-OFF regimen. The cell

number was readjusted to $0.4x10^6$ cells/ml every 24 hours. Data are shown as mean ± SD of n = 3 biologically independent samples, representative of n = 3 independent experiments. In panels **a, b** individual data points are shown as dots and statistical significance was calculated by one-way ANOVA and two-way ANOVA, respectively. ns: not significant, $^{**}P < 0.01$, $^{***}P < 0.001$, $^{****}P < 0.0001$.

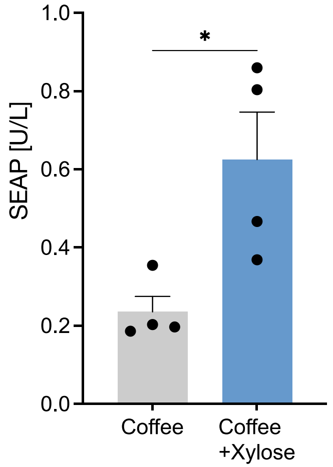

Figure S4. Blood SEAP levels in response to xylose-sweetened coffee. Wild-type mice implanted with encapsulated SWEET-engineered cells received either coffee or xylose-sweetened coffee (0.5 g/kg, o.g.). Data are shown as mean ± SEM of n = 4 mice (the individual data points are shown as dots). Statistical significance was calculated by unpaired t-test (versus untreated group of mice). $^{*}P < 0.05$.

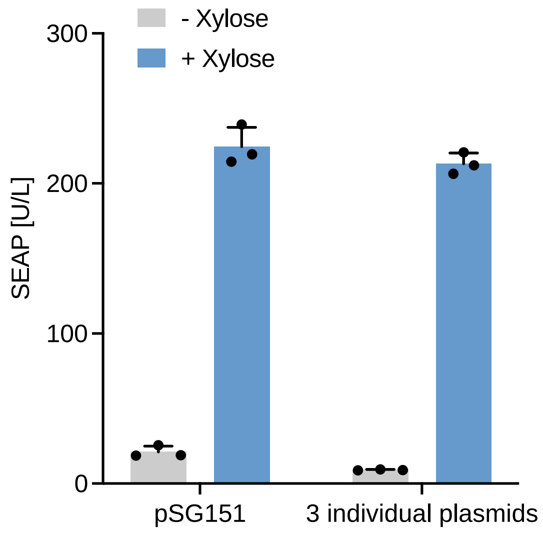

Figure S5. Validation of the single plasmid encoding all three SWEET components. HEK293T cells were transfected with either 75 ng of pSG151 and 75 ng of empty plasmid or with 50 ng of pOM219, pSG098 and pSG126. SEAP was measured 24 hours after incubation in medium without or with xylose (250 µM). Data are shown as mean ± SD of n = 3 biologically independent samples, representative of n = 3 independent experiments (the individual data points are shown as dots).

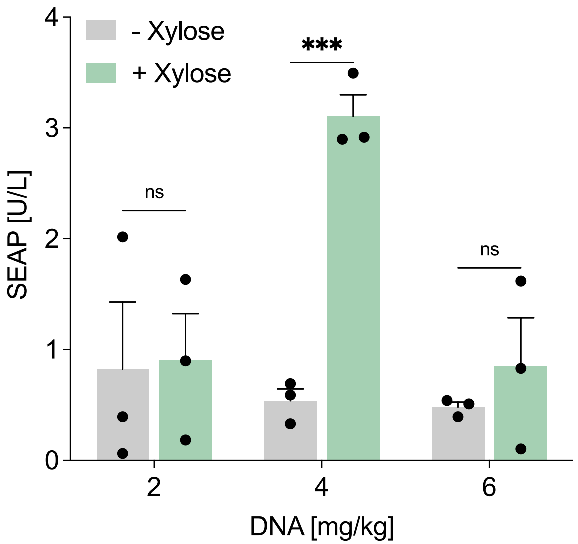

Figure S6. Effect of DNA dosage for hydrodynamic transfection. Wild-type mice injected with the indicated amounts of pSG151 were supplemented with 1 g/kg of xylose by o.g.. Blood samples for SEAP profiling were withdrawn 24 hours after induction. Data are shown as mean ± SEM of n = 3 mice (the individual data points are shown as dots). Statistical significance was calculated by two-way ANOVA. ns: not significant, ***P < 0.001.

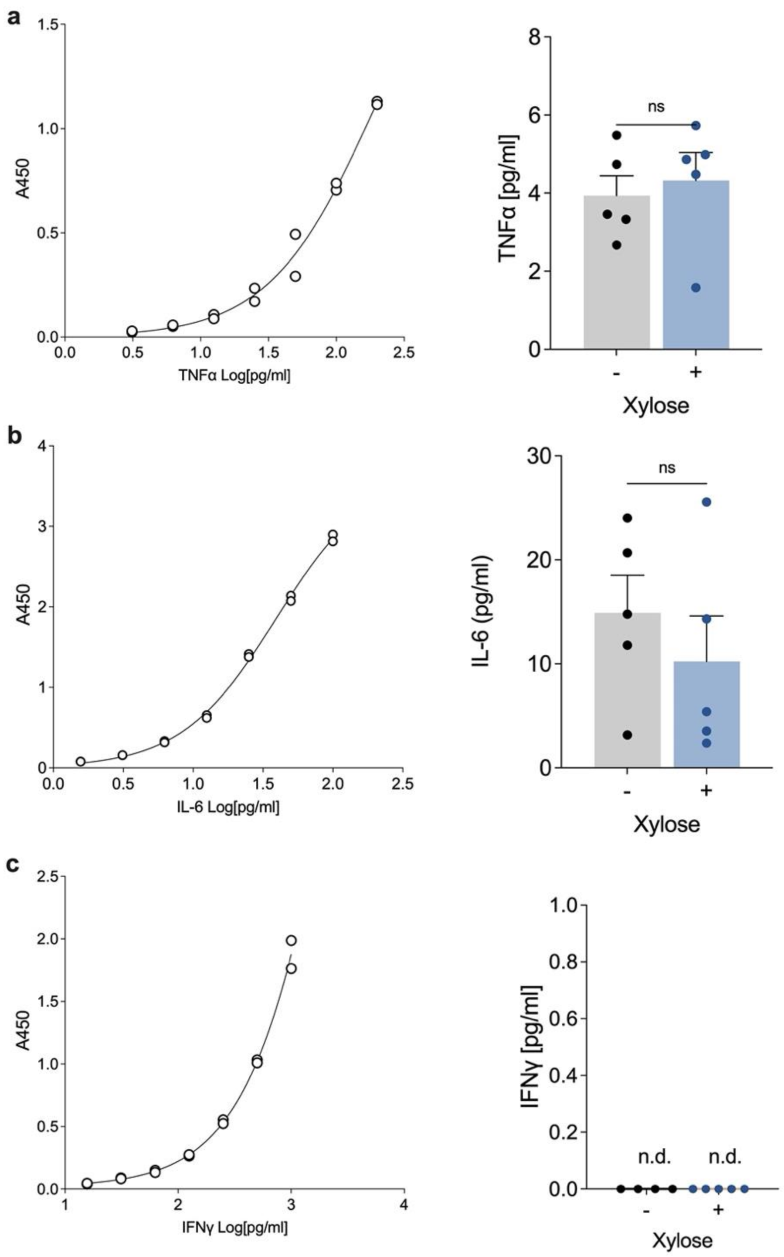
a
A450
TNFα Log[pg/ml]
TNFα [pg/ml]
ns
Xylose
b
A450
IL-6 Log[pg/ml]
IL-6 (pg/ml)
ns
Xylose
c
A450
IFNγ Log[pg/ml]
IFNγ [pg/ml]
n.d.
n.d.
Xylose

Figure S7. Analysis of inflammatory cytokines in serum of SWEET-engineered mice. Wild-type mice were injected with pSG151 and either treated or not treated with xylose (1 g/kg), once per week for two consecutive weeks before blood withdrawal and analysis of **a** TNFα, **b** IL-6, **c** IFNγ. The cytokines were measured using specific ELISA kits. The left panels show the calibration curves calculated using a 4-parameter curve fit, and the right panels show the values acquired for each cytokine in untreated and treated SWEET-engineered mice. Data are shown as mean ± SEM of n = 5 mice (the individual data points are shown as dots). Statistical significance was calculated by unpaired t-test. ns: not significant, n.d.: not detectable.

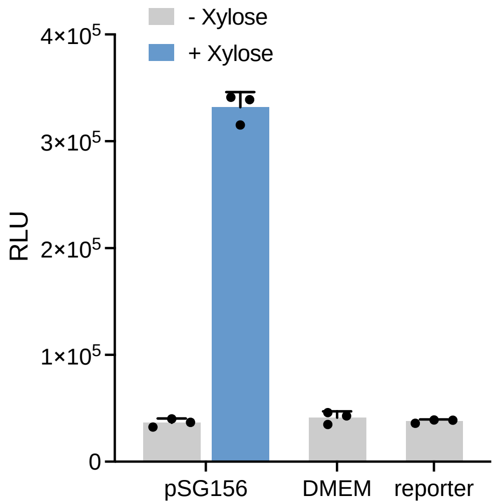

Figure S8. In vitro validation of SWEET$_{ins}$ (pSG156) for the production of insulin. HEK293T cells were transfected with pSG156 and cultivated in medium without or with xylose (1 mM). After 24 hours, the supernatant was taken and added to HEK293T cells expressing the insulin receptor (pIR), a NanoLuc reporter plasmid (pLeo665) and Elk1-TetR (MKp37) fusion protein (Keeley et al., 2005). When insulin binds the

insulin receptor, it initiates a signaling cascade that ultimately leads to activation of MAPK pathway and phosphorylation of Elk1-TetR, which promotes NanoLuc expression from O_{TetO7} promoter. Controls are cells cultivated in DMEM only (DMEM control) or cells transfected with MKp37, pLeo665 and a mock plasmid (reporter control). Data are shown as mean ± SD of n = 3 biologically independent samples induced from n = 3 independent samples, representative of n = 3 independent experiments (the individual data points are shown as dots).

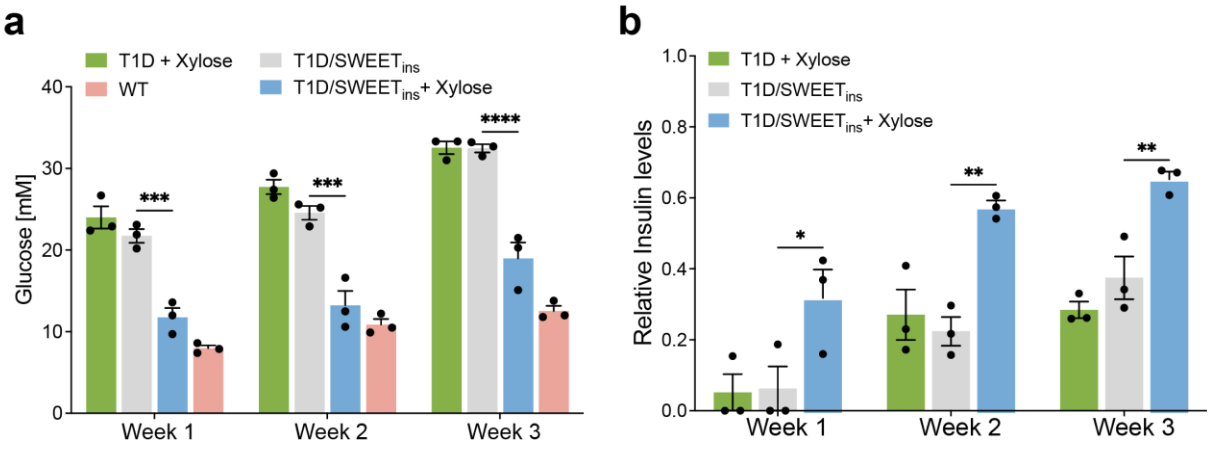

Figure S9. Therapeutic output of $SWEET_{ins}$ in T1D mice up to three weeks. **a** Fasting blood glucose and **b** insulin levels of $SWEET_{ins}$-engineered T1D mice. Mice were supplemented or not with xylose (0.5 g/kg) by o.g. once per week for three consecutive weeks. Insulin is expressed as relative levels to that of the wild-type mice, from 0 = below detection threshold, to 1 = average insulin level of wild-type mice for the given time point. Data are shown as mean ± SEM of n = 3 mice (the individual data points are shown as dots). Statistical significance was calculated by two-way ANOVA between treated and untreated groups. *$P < 0.05$, **$P < 0.01$, ***$P < 0.001$, ****$P < 0.0001$.

Table S1 | Plasmids used in this work

**Plasmid ID**	**Detailed Cloning Description**	**Source**
pIR	Constitutive human insulin receptor expression vector (P_{hCMV}-IR-pA_{bGH})	Jacob et al., 2002 Addgene (no.24049)
pJH1040	P_{hCMV}-driven expression of SEAP and mINS (P_{hCMV}-SEAP-p2A-mINS-pA_{bGH}).	Huang et al., unpublished
pLeo665	Mammalian reporter plasmid encoding O_{TetO7}-$P_{hCMVmin}$ driven expression of secreted NanoLuc reporter protein (O_{TetO7}-$P_{hCMVmin}$-ss-NLuc-pA_{bGH}).	Scheller et al, 2018
MKp37	Constitutive mammalian TetR-ELK1 fusion protein expression vector (P_{hPGK}-TetR-ELK1-pA_{SV40})	Keeley et al. 2018
pTS395	Vector coding for P_{hCMV}-driven Sleeping Beauty transposase mammalian expression vector (P_{hCMV}-SB100-pA_{bGH}).	Haellman et al., 2021
pTS1022	Vector encoding P_{hCMV}-driven SEAP expression cassette (P_{hCMV}-SEAP-pA_{bGH}).	Haellman et al., 2021
pTS1107	Tier-3 vector for stable integration of up to three cassettes via Sleeping Beauty transposase (5'ITR-cassette1-pA_{bGH}::cassette2-pA_{p36}::cassette3-pA_{p9}-3'ITR).	Haellman et al., 2021
pTS1029	Tier-3 vector for stable integration of up to two cassettes via Sleeping Beauty transposase, encodes YPet-p2A-PuroR for selection. (5'ITR-cassette1-pA_{bGH}::cassette2-pA_{p36}::P_{RPBSA}-YPet-p2A-PuroR - 3'ITR).	Haellman et al., 2021
pTS1214	P_{hCMV}-driven expression vector with self-cleaving p2A peptide between MCSs (P_{hCMV}-MCS-p2A-MCS-pA_{bGH}).	Strittmatter et al. unpublished
pTS2367	Vector encoding P_{hCMV}-driven VP16 expression cassette (P_{hCMV}-VP16-pA_{bGH}).	Strittmatter et al.

		unpublished
pTS2368	Vector encoding P_{hCMV}-driven VP64 expression cassette (P_{hCMV}-VP64-pA_{bGH}).	Strittmatter et al. unpublished
pVH15	Vector encoding P_{hCMV}-driven VPR expression cassette (P_{hCMV}-VPR-pA_{bGH}).	Haellman et al., 2021
pOM211	Reporter vector encoding SEAP under the control of a XylR-responsive promoter consisting of the IA binding site upstream of a minimal promoter ($XylO_{IA}$-P_{min}-SEAP-pA_{bGH}). The $XylO_{IA}$ sequence was assembled by annealing of the oligos oOM296 and oOM297 and ligated into pOM213 restricted with XhoI/PacI.	This work
pOM212	Reporter vector encoding SEAP under the control of a XylR-responsive promoter consisting of the IF binding site upstream of a minimal promoter ($XylO_{IF}$-P_{min}-SEAP-pA_{bGH}). The $XylO_{IF}$ sequence was assembled by annealing of the oligos oOM298 and oOM299 and ligated into pOM213 restricted with XhoI/PacI.	This work
pOM213	Reporter vector encoding SEAP under the control of a XylR-responsive promoter consisting of the native *E. coli* XylR-binding region (IA-108bp-IF; $XylO_{wt}$) upstream of a minimal promoter ($XylO_{wt}$-P_{min}-SEAP-pA_{bGH}). The synthetic promoter ($XylO_{wt}$-P_{min}) was synthesized by Twist Bioscience, digested with XhoI/EcoRI and ligated into pTS1022 restricted with XhoI/EcoRI.	This work
pOM214	Vector coding for a constitutive XylR-VP16 expression cassette (P_{hCMV}-XylR-VP16-pA_{bGH}). A human codon-optimized version of XylR from *E. coli* was synthesized by Twist Bioscience, digested with SpeI/NheI and ligated into pTS2367 restricted with SpeI.	This work
pOM215	Vector coding for a constitutive XylR-VP64 expression cassette (P_{hCMV}-XylR-VP64-pA_{bGH}). A human codon-optimized version of XylR from *E. coli* was synthesized by Twist Bioscience,	This work

	digested with SpeI/NheI and ligated into pTS2368 restricted with SpeI.	
pOM216	Vector coding for a constitutive XylR-VPR expression cassette (P_{hCMV}-XylR-VPR-pA_{bGH}). A human codon-optimized version of XylR from *E. coli* was synthesized by Twist Bioscience, digested with SpeI/NheI and ligated into pVH15 restricted with SpeI.	This work
pOM217	Vector coding for a constitutive XylR-VP16 expression cassette with a NLS at the N-terminus of XylR (P_{hCMV}-NLS-XylR-VP16-pA_{bGH}). The SV40 NLS was assembled by annealing of the oligos oAPT265 and oAPT266 and ligated into pOM214 restricted with EcoRI/SpeI.	This work
pOM218	Vector coding for a constitutive XylR-VP64 expression cassette with a NLS at the N-terminus of XylR (P_{hCMV}-NLS-XylR-VP64-pA_{bGH}). The SV40 NLS was assembled by annealing of the oligos oAPT265 and oAPT266 and ligated into pOM215 restricted with EcoRI/SpeI.	This work
pOM219	Vector coding for a constitutive XylR-VPR expression cassette with a NLS at the N-terminus of XylR (P_{hCMV}-NLS-XylR-VP64-pA_{bGH}). The SV40 NLS was assembled by annealing of the oligos oAPT265 and oAPT266 and ligated into pOM216 restricted with EcoRI/SpeI.	This work
pOM232	Vector coding for a constitutive VP16-XylR expression cassette (P_{hCMV}-VP16-XylR-pA_{bGH}). XylR was PCR amplified from pOM214 with the oligos oOM294 and oOM295, digested with SgrAI/HindIII and ligated into pTS2367 restricted with SgrAI/HindIII.	This work
pOM233	Vector coding for a constitutive VP64-XylR expression cassette (P_{hCMV}-VP64-XylR-pA_{bGH}). XylR was PCR amplified from pOM214 with the oligos oOM294 and oOM295, digested with SgrAI/HindIII and ligated into pTS2368 restricted with SgrAI/HindIII.	
pOM234	Vector coding for a constitutive VPR-XylR expression cassette (P_{hCMV}-VPR-XylR-pA_{bGH}). XylR was PCR amplified from pOM214 with the oligos oOM294 and oOM295, digested with	This work

	SgrAI/HindIII and ligated into pVH15 restricted with SgrAI/HindIII.	
pOM235	Vector coding for a constitutive VP16-XylR expression cassette with a NLS at the N-terminus of VP16 (P_{hCMV}-NLS-VP16-XylR-pA_{bGH}). The SV40 NLS was assembled by annealing of the oligos oAPT265 and oAPT266 and ligated into pOM232 restricted with EcoRI/SpeI.	This work
pOM236	Vector coding for a constitutive VP64-XylR expression cassette with a NLS at the N-terminus of VP64 (P_{hCMV}-NLS-VP64-XylR-pA_{bGH}). The SV40 NLS was assembled by annealing of the oligos oAPT265 and oAPT266 and ligated into pOM233 restricted with EcoRI/SpeI.	This work
pOM237	Vector coding for a constitutive VPR-XylR expression cassette with a NLS at the N-terminus of VPR (P_{hCMV}-NLS-VPR-XylR-pA_{bGH}). The SV40 NLS was assembled by annealing of the oligos oAPT265 and oAPT266 and ligated into pOM234 restricted with EcoRI/SpeI.	This work
pOM247	Reporter vector coding for SEAP reporter protein under the control of a XylR-responsive promoter consisting of the IA and IF binding sites separated by 13 bp, which are the 13 bp downstream of IA in the native promoter (IA-13bp-IF; $XylO_{13bp}$) upstream of a minimal promoter ($XylO_{13bp}$-P_{min}-SEAP-pA_{bGH}). The $XylRO_{13bp}$ region was assembled by annealing of oligos oOM290 and oOM291, and ligated into pOM213 restricted with XhoI/PacI.	This work
pOM248	Reporter vector coding for SEAP reporter protein under the control of a XylR-responsive promoter consisting of the IA and IF binding sites separated by 25 bp, which are the 25 bp downstream of IA in the native promoter (IA-25bp-IF; $XylO_{25bp}$) upstream of a minimal promoter ($XylO_{25bp}$-P_{min}-SEAP-pA_{bGH}). The $XylRO_{25bp}$ region was assembled by annealing of oligos oOM292 and oOM293, and ligated into pOM213 restricted with XhoI/PacI.	This work
pOM249	Reporter vector coding for SEAP reporter protein under the control of a XylR-responsive promoter	This work

	consisting of the IA and IF binding sites separated by 37 bp, which are the 37 bp downstream of IA in the native promoter (IA-37bp-IF; $XylO_{37bp}$) upstream of a minimal promoter ($XylO_{37bp}$-P_{min}-SEAP-pA_{bGH}). The $XylO_{37bp}$ region was assembled by annealing of four oligos (oOM300, oOM301, oOM302 and oOM303), and ligated into pOM213 restricted with XhoI/PacI.	
pOM250	Reporter vector coding for SEAP reporter protein under the control of a XylR-responsive promoter consisting of the IA and IF binding sites separated by 49 bp, which are the 49 bp downstream of IA in the native promoter (IA-49bp-IF; $XylO_{49bp}$) upstream of a minimal promoter ($XylO_{49bp}$-P_{min}-SEAP-pA_{bGH}). The $XylO_{49bp}$ region was assembled by annealing of four oligos (oOM304, oOM305, oOM306 and oOM307), and ligated into pOM213 restricted with XhoI/PacI.	This work
pSG089	Reporter vector coding for SEAP reporter protein under the control of a XylR-responsive promoter consisting of two tandem repeats of $XylO_{49bp}$ (IA-49bp-IF-42bp-IA-49bp-IF; $XylO_{49bp(2x)}$) separated by a randomly generated, 42 bp long sequence upstream of a minimal promoter ($XylO_{49bp(2x)}$-P_{min}-SEAP-pA_{bGH}). The construct was cloned by Gibson assembly. The $XylO_{49bp}$ sequence was PCR amplified with oligos oSG082 and oSG083 from pOM250; the backbone was amplified with oligos oSG84-oSG85 from pOM250.	This work
pSG098	Reporter vector coding for SEAP reporter protein under the control of a XylR-responsive promoter consisting of three tandem repeats of $XylO_{49bp}$ (IA-49bp-IF-42bp-IA-49bp-IF-42bp-IA-49bp-IF; $XylO_{49bp(3x)}$) separated by two randomly generated, 42 bp long DNA sequences upstream of a minimal promoter ($XylO_{49bp(3x)}$-P_{min}-SEAP-pA_{bGH}). The construct was cloned by Gibson assembly. The $XylO_{49bp}$ sequence was PCR amplified with oligos oSG104 and oSG082 from pSG089; the backbone was amplified with oligos oSG104-oSG85 from pOM250.	This work

pSG125	Vector coding for a constitutive $Gal2_{N376F}$ expression cassette (P_{hCMV}-$Gal2_{N376F}$-pA_{bGH}). A human codon-optimized version of $Gal2_{N376F}$ from *S. cerevisiae* was synthesized by Twist Bioscience, digested with EcoRI/HindIII and ligated into pTS1022 restricted with EcoRI/HindIII.	This work
pSG126	Vector coding for a constitutive $Xltr1p_{N32F}$ expression cassette (P_{hCMV}- $Xltr1p_{N32F}$-pA_{bGH}). A human codon-optimized version of $Xltr1p_{N32F}$ from *T. reesei* was synthesized by Twist Bioscience, digested with EcoRI/HindIII and ligated into pTS1022 restricted with EcoRI/HindIII.	This work
pSG145	Tier-3 vector with YPet and PuroR and a cassette coding for SEAP reporter protein under the control of $XylO_{49bp(3x)}$ ($XylO_{49bp(3x)}$-P_{min}-SEAP-pA_{p36}). $XylO_{49bp(3x)}$-P_{min}-SEAP was restricted from pSG098 with MluI/HindIII and ligated into pTS1029 restricted with BsaI.	
pSG146	Vector coding for p2A-$Xltr1p_{N326F}$ under the control of the constitutive P_{hCMV} promoter (P_{hCMV}-p2A-$Xltr1p_{N326F}$-pA_{bGH}). $Xltr1p_{N326F}$ was isolated from pSG126 by restriction digestion with SpeI/HindIII and ligated into pTS1214 restricted with NheI/HindIII.	This work
pSG147	Vector coding for XylR-VPR and $Xltr1p_{N326F}$ separated by p2A and under the control of the constitutive P_{hCMV} promoter (P_{hCMV}-NLS-XylR-VPR-p2A-$Xltr1p_{N326F}$-pA_{bGH}). pSG146 was digested with EcoRI/SpeI for the insertion of NLS-XylR-VPR, restricted from pOM219 with EcoRI/NheI.	This work
pSG148	Tier-3 vector with a cassette coding for SEAP reporter protein under the control of $XylO_{49bp(3x)}$ ($XylO_{49bp(3x)}$-P_{min}-SEAP-pA_{p36}). $XylO_{49bp(3x)}$-P_{min}-SEAP was restricted from pSG098 with MluI/HindIII and ligated into pTS1107 restricted with BsaI.	This work
pSG149	Tier-3 plasmid with YPet fluorophore, puromycin selection, and containing all the SWEET components. The first cassette codes for NLS-XylR-VPR and $Xltr1p_{N326F}$, which are separated by p2A sequence and expressed with the constitutive P_{hCMV} promoter (P_{hCMV}-NLS-XylR-VPR-p2A-	This work

	Xltr1p$_{N326F}$-pA$_{bGH}$), followed by a cassette coding for SEAP reporter protein under the control of XylO$_{49bp(3x)}$ (XylO$_{49bp(3x)}$-P$_{min}$-SEAP-pA$_{p36}$). The first cassette was isolated from pSG147 by MluI/HindIII restriction digestion and ligated into pSG145 restricted with MluI/HindIII.	
pSG151	Tier-3 plasmid containing all the SWEET components. The first cassette codes for NLS-XylR-VPR and Xltr1p$_{N326F}$, which are separated by p2A sequence and expressed with the constitutive P$_{hCMV}$ promoter (P$_{hCMV}$-NLS-XylR-VPR-p2A-Xltr1p$_{N326F}$-pA$_{bGH}$), followed by a cassette coding for SEAP reporter protein under the control of XylO$_{49bp(3x)}$ (XylO$_{49bp(3x)}$-P$_{min}$-SEAP-pA$_{p36}$). The first cassette was isolated from pSG147 by MluI/HindIII restriction digestion and ligated into pSG148 restricted with MluI/HindIII.	This work
pSG154	Vector coding for SEAP-p2A-mINS under the control of XylO$_{49bp(3x)}$ (XylO$_{49bp(3x)}$-P$_{min}$-SEAP-p2A-mINS-pA$_{bGH}$). XylO$_{49bp(3x)}$ was isolated from pSG098 by digestion with MluI/EcoRI and inserted into pJH1040 restricted with MluI/EcoRI.	This work
pSG155	Tier-3 vector with a cassette coding for SEAP-p2A-mINS under the control of XylO$_{49bp(3x)}$ (XylO$_{49bp(3x)}$-P$_{min}$-SEAP-p2A-mINS-pA$_{p36}$). XylO$_{49bp(3x)}$-P$_{min}$-SEAP-p2A-mINS was restricted from pSG154 with MluI/HindIII and ligated into pTS1107 restricted with BsaI.	This work
pSG156	Vector containing all the SWEET$_{ins}$ components. The first cassette codes for NLS-XylR-VPR and Xltr1p$_{N326F}$, which are separated by p2A sequence and expressed with the constitutive P$_{hCMV}$ promoter (P$_{hCMV}$-NLS-XylR-VPR-p2A-Xltr1p$_{N326F}$-pA$_{bGH}$), followed by a cassette coding for SEAP-p2A-mINS under the control of XylO$_{49bp(3x)}$ (XylO$_{49bp(3x)}$-P$_{min}$-SEAP-p2A-mINS-pA$_{p36}$). The first cassette was isolated from pSG147 by MluI/HindIII restriction digestion and ligated into pSG155 restricted with MluI/HindIII.	This work

pSG164	Reporter vector encoding SEAP reporter protein under the control of a XylR-responsive promoter consisting of two IA sites separated by the native 108 bp spacing region (IA-108bp-IA; $XylO_{IA-IA}$) upstream of a minimal promoter ($XylO_{IA-IA}$-P_{min}-SEAP-pA_{bGH}). The construct was cloned by performing mutagenesis PCR on pOM213 amplified with oligos oSG182-oSG183.	This work
pSG165	Reporter vector encoding SEAP reporter protein under the control of a XylR-responsive promoter consisting of two IF sites separated by the native 108 bp spacing region (IF-108bp-IF; $XylO_{IF-IF}$) upstream of a minimal promoter ($XylO_{IF-IF}$-P_{min}-SEAP-pA_{bGH}). The construct was cloned by performing mutagenesis PCR on pOM213 amplified with oligos oSG184-oSG185.	This work
pSG166	Reporter vector encoding SEAP reporter protein under the control of a XylR-responsive promoter consisting of IA and IA_{rev} sites separated by the native 108 bp spacing region (IA-108bp-IA_{rev}; $XylO_{IA-IArev}$) upstream of a minimal promoter ($XylO_{IA-IArev}$-P_{min}-SEAP-pA_{bGH}). The construct was cloned by performing mutagenesis PCR on pOM213 amplified with oligos oSG186-oSG187.	This work
pSG167	Reporter vector encoding SEAP reporter protein under the control of a XylR-responsive promoter consisting of IF_{rev} and IF sites separated by the native 108 bp spacing region (IF_{rev}-108bp-IF; $XylO_{IFrev-IF}$) upstream of a minimal promoter ($XylO_{IFrev-IF}$-P_{min}-SEAP-pA_{bGH}). The construct was cloned by performing mutagenesis PCR on pOM213 amplified with oligos oSG188-oSG189.	This work

Abbreviations and additional information

ELKI, ETS transcription factor ELK1; **$Gal2_{N376F}$**, mutated version of xylose transporter from *S. cerevisiae*; **IA**, XylR binding domain in XylO; **IArev**, reverse complement of IA; **IF**, XylR binding domain in XylO; **IFrev**, reverse complement of IF; **IR**, insulin receptor; **MCS**, multiple cloning site; **mINS**, insulin variant optimized for production in HEK293T cells; **NLS**, nuclear localization sequence; **NLuc**, NanoLuc luciferase; **O_{TetO7}**, heptameric TetR-specific operator; **p2A**, 2A self-cleaving peptide; **pA_{bGH}**, polyadenylation signal from the bovine growth hormone;

pA_{p36}, synthetic polyadenylation signal; **pA_{SV40}**, polyadenylation signal from simian virus 40; **P_{hCMV}**, human cytomegalovirus immediate-early promoter; **P_{min}**, pGL4.23-derived minimal promoter; **P_{hPGK}**, human 3-phosphoglycerate kinase promoter; **PuroR**, puromycin resistance gene; **SEAP**, human placental secreted alkaline phosphatase; **ss**, signal sequence; **TetR**, *E. coli* Tn10-derived tetracycline-dependent repressor; **VP16**, herpes simplex virus-derived transactivation domain; **VP64**, four tandem repeats of VP16; **VPR**, fusion of the three transactivator domains VP64-p65-Rta; **$Xltr1p_{N326F}$**, mutated version of xylose transporter from *T. reesei*; **XylO**, xylose operator for XylR binding; **XylR**, xylose operon regulatory protein from *E. coli*; **YPet**, modified yellow fluorescent protein.

Table S2 | Oligonucleotide sequences used in this work

**Oligo ID**	**Oligonucleotide Sequence (5'-3')**
oAPT265	5'- aattcaccatgccaaagaaaaagaggaaagtca -3'
oAPT266	5'- ctagtgactttcctctttttctttggcatggtg -3'
oOM290	5'-tcgagcaattatgttatttcacactgctattgagataattcacaagtgtgcgctcgctcgcaagaaataaaccaaaaatcgtaatcgaaagataaaaatctgttaat-3'
oOM291	5'-taacagatttttatctttcgattacgatttttggtttatttcttgcgagcgagcgcacacttgtgaattatctcaatagcagtgtgaaataacataattgc-3'
oOM292	5'tcgagcaattatgttatttcacactgctattgagataattcacaagtgtgcgctcgctcgcaaaataaaatgcaagaaataaaccaaaaatcgtaatcgaaagataaaaatctgttaat-3'
oOM293	5'-TAAcagatttttatctttcgattacgatttttggtttatttcttgcattttattttgcgagcgagcgcacacttgtgaattatctcaatagcagtgtgaaataacataattgC-3'
oOM294	5'-cagcagcaccggtggtttcactaagagacaccgcatc-3'
oOM295	5'-cagcagaagcttgagcatcacctctgagttcac-3'
oOM296	5'-tcgagcaattatgttatttcacactgctattgagataattcacaagtgttaat-3'

oOM297	5'-taacacttgtgaattatctcaatagcagtgtgaaataacataattgc-3'
oOM298	5’-tcgagcaagaaataaaccaaaaatcgtaatcgaaagataaaaatctgttaat-3’
oOM299	5'-taacagatttttatctttcgattacgatttttggtttatttcttgc-3'
oOM300	5’-tcgagcaattatgttatttcacactgctattgagataattcacaagtgtgcgctcgctcgcaaaataaaatggaatgatgaaaccaag-3’
oOM301	5’-aaataaaccaaaaatcgtaatcgaaagataaaaatctgttaat-3’
oOM302	5'-acttgtgaattatctcaatagcagtgtgaaataacataattgc-3'
oOM303	5’-taacagatttttatctttcgattacgatttttggtttatttcttggtttcatcattccattttattttgcgagcgagcgcac-3'
oOM304	5’-tcgagcaattatgttatttcacactgctattgagataattcacaagtgtgcgctcgctcgcaaaataaaatggaatgatgaaactgg-3’
oOM305	5’-gtaattcctcaagaaataaaccaaaaatcgtaatcgaaagataaaaatctgttaat-3’
oOM306	5'-gtaattcctcaagaaataaaccaaaaatcgtaatcgaaagataaaaatctgttaat-3'
oOM307	5'-taacagatttttatctttcgattacgatttttggtttatttcttgaggaattacccagtttcatcattccattttattttgcgag -3'
oSG082	5’-agatctccacgcgtggtaccctcgagcaattatgttatttcacactgc-3’
oSG083	5’- agatctccacgcgtggtaccctcgagcaattatgttatttcacactgc-3’
oSG084	5’-cggtgatgagaagtatgcatctcgttccccaattatgttatttcacactgct-3’
oSG085	5’- taccacgcgtggagatct-3’
oSG104	5’-tagctccggcaagcaattaagtaccctcgagcaattatgt-3’
oSG105	5’-attgcttgccggagctaaagacgttccacagatttttatctttcgattacg-3’
oSG182	5’-atctcaatagcagtgtgaaataacataattgatttatgaccgagatcttac-3’
oSG183	5’-actgctattgagataattcacaagtttaattaatagagggtatataatgg-3’
oSG184	5’-tttcgattacgatttttggtttatttcttgctcgagggtaccacgcgt-3’
oSG185	5’-aatcgtaatcgaaagataaaaatctggtgcgctcgctcgcaaaata-3’

oSG186	5’-tttcacactgctattgagataattcacaagtatttatgaccgagatcttac-3’
oSG187	5’-atagcagtgtgaaataacataattgttaattaatagagggtatataatgg-3’
oSG188	5’-caaaaatcgtaatcgaaagataaaaatctgctcgagggtaccacgcgt-3’
oSG189	5’-gattacgatttttggtttatttcttggtgcgctcgctcgcaaaata-3’

Table S3 | Codon-optimized gene fragments used in this work

**Gene name**	**Gene fragment sequence (5’-3’)**
Gal2$_{N376F}$	CTGTTCGAAGCGGAATTCACCATGACTAGTGCAGTTGAGGAGAACAACATGCCCGTGGTGTCACAACAACCTCA GGCAGGGGAAGACGTTATCTCTAGCTTATCTAAGGATTCCCACCTCAGCGCCCAATCACAAAAGTATAGCAATG ATGAGCTCAAAGCCGGAGAGTCTGGTAGTGAAGGCAGTCAATCTGTGCCTATCGAGATCCCAAAGAAACCTATG TCTGAGTATGTCACCGTGTCTCTGCTTTGTCTGTGTGTGGCATTTGGCGGATTTATGTTCGGCTGGGACACCGGAA CGATCAGCGGCTTCGTAGTGCAGACCGATTTCCTGAGGCGCTTCGGGATGAAACACAAAGACGGAACACACTAC TTATCTAACGTCAGGACTGGGCTGATTGTCGCCATCTTTAATATTGGATGTGCATTTGGCGGTATCATCCTGAGTA AGGGTGGGGATATGTATGGTAGGAAGAAGGGCCTCAGTATTGTCGTGTCTGTGTATATTGTGGGGATCATCATAC AAATTGCATCAATAAACAAGTGGTATCAGTACTTTATTGGACGGATTATTAGTGGGCTGGGTGTCGGTGGAATCG CCGTCCTGTGCCCAATGTTAATTAGTGAAATCGCTCCGAAGCATCTGCGGGGAACCTTGGTGAGTTGTTACCAGC TGATGATTACTGCCGGGATCTTTCTTGGCTATTGTACTAATTATGGGACTAAATCCTATTCCAATAGCGTCCAGTG GCGCGTGCCGCTGGGGTTATGCTTCGCTTGGTCCCTTTTCATGATTGGCGCTCTGACCCTTGTGCCTGAGTCACCC CGCTACCTCTGTGAAGTCAACAAGGTCGAAGACGCCAAGCGGTCTATCGCCAAAAGCAATAAAGTCAGCCCAGA GGATCCTGCGGTTCAAGCCGAGCTGGATCTGATTATGGCGGGCATCGAAGCAGAAAAGTTAGCAGGGAATGCTT CTTGGGGCGAGTTATTCAGTACCAAAACGAAAGTTTTCCAACGCTTACTGATGGGCGTGTTTGTGCAGATGTTTC

	AACAGTTGACAGGGAACAACTATTTCTTTTATTATGGAACTGTTATTTTCAAGTCTGTGGGGCTGGACGATTCCTT<br>TGAGACTAGCATTGTAATTGGCGTCGTCTTCTTTGCTAGTACGTTCTTTTCCTTGTGGACAGTGGAGAACCTCGGA<br>CATAGAAAGTGCCTGCTGCTCGGAGCCGCAACTATGATGGCGTGTATGGTGATTTACGCATCAGTCGGAGTCACT<br>AGACTGTATCCCCATGGGAAGTCTCAACCAAGCTCAAAAGGCGCCGGCAACTGCATGATTGTCTTCACTTGCTTC<br>TACATTTTCTGCTATGCAACCACCTGGGCACCAGTGGCTTGGGTAATCACCGCCGAGTCCTTCCCTCTGCGTGTGA<br>AGAGCAAGTGTATGGCCTTAGCCTCAGCCAGCAACTGGGTCTGGGGCTTCCTGATAGCTTTCTTCACTCCATTTAT<br>AACTTCAGCAATTAATTTCTATTACGGCTACGTATTCATGGGATGTCTGGTGGCTATGTTCTTTTATGTGTTCTTCT<br>TCGTGCCAGAAACGAAGGGCCTCTCACTGGAAGAGATCCAAGAACTCTGGGAAGAAGGAGTGCTGCCCTGGAAG<br>TCAGAGGGGTGGATACCTAGCTCAAGACGCGGTAATAACTACGACCTGGAAGACCTGCAACACGACGATAAGCC<br>TTGGTACAAAGCCATGTTGGAAGCTAGCGGATCCACCGGTGTCTAGAAAGCTTTGAGGCCGGCCT
Xltr1p$_{N326F}$	CTGTTCGAAGCGGAATTCACCATGACTAGTCGATTCAGTGAGAAACTTGGGTTTAAGCGACCTGACGATGAGGCC<br>GGGGCATCATGGGTGGCGATCCTGATGGGATTCTTCGTCGCTTTCGGCGGAGTCCTGTATGGTTATGACACTGGA<br>ACAATCAGCGGAATAATGGCAATGCCATATTTCAAAGATTTGTTCTCCACTGGCTATAGGAACCCTAATGGCGAA<br>TTAGATATCACTGCCACTCAAGAAAGCGCCATAGTATCTATTCTCTCCGCAGGGACCTTCTTTGGGGCTTTGGCGT<br>CTCCGCTTCTGGCTGATTTTCTCGGTCGAAGGCCCGCCCTGATGATCAGTACGTGGGTCTTTAACCTGGGCGTAGT<br>CTTACAAACTATCGCCACTGCAATCCCTATGTTCCTGGCAGGACGGTTCTTCGCCGGGTTCGGGGTAGGACTGAT<br>AAGTGCACTCATTCCTCTGTATCAGTCTGAGACTGCGCCTAAATGGATACGGGGCGCCATTGTTGGGGCGTATCA<br>GCTGGCGATTACAATCGGGCTTCTGCTTGCAGCTGTGGTAAATAACGCTACTGCCAAACGCCACGATTCAGGGTC<br>ATACCGCATCCCAATTGCAGTCCAGTTTGCCTGGTCCCTTGTCTTGTTCGTTGGCATGATCTTTCTGCCCGAGACT<br>CCTAGGTTTCTCGTTAGGTCCGGAAAACTCGAAAAGGCACGCGCAGCCCTGAGCCGGATACGCAGACTGTCCCC<br>CGAGCACGAAGCCTTGGCGGCAGAACTTGGTCAAATCCAGGCCAATCTGGAAGCTGAAAGCAGCGTACGGAAG

	GCCACATATGCCGATTGCTTTCGCAGGCCTATGCTGAAGCGCCAATTCACCGGAATGGCTCTCCAAGCCTTACAG<br>CAATTGACAGGAATCAATTTCATCTTTTATTATGGCACCAGGTACTTCCAAAACAGTGGGGTGTCCAGCGGCTTC<br>ACTATCGGCATGATTACAGCGGGTATATTCGTGGCGTCTACAATCCCCGGACTCCTCGCCATTGACCGTTGGGGT<br>CGCAGGCCCCTGCTGCTGCTGGGTGCGGTAGGAATGTGTGTGTCACAGCTGATTGTGGCCGTCGTTGGTACCGTT<br>TCCACTGGCCAAAGGCCAAACGGGGAAATCTTCGTTAAATCCTTGGCTGGGCAGCAGGCAGCCGTGGCCTTTGTA<br>TGTATCTTTATTGCATTCTTTGCAAGCACCTGGGGTCCACTCGCATGGGTGGTCACCGGGGAGATCTACCCACTG<br>GCCACTCGGGCTAAAGCACTCTCCATGACAACTGCTACCAACTGGCTGTTTAATTGGGCCATTGCCTACAGCACA<br>CCCTATCTGGTCAACTACGGCCCTGGGTACGCCAATTTGCAGAGCAAGATATTCTTTGTTTGGTTCGGTGCATGCT<br>TCTTGTGCATAGCTTTAGTGTGGTTCTTCATCTACGAGACAAAAGGTTTGTCCCTGGAAGAAGTGGACGAGCTTT<br>ATGCCGAGGTCAAAGTCGCCCGCAAATCAACTACTTGGAAGCCGACCCCACGGCTGGAAGCGGCTGGGTCTACA<br>ACCAGCGAGGAATCAAAGGACGAAAGTGGCCCAAAGGAAGCATCCCCACACGTAATGGATCAAGGCGTGGAAC<br>TGCAAGTGGCTAGCGGATCCACCGGTGTCTAGAAAGCTTTGAGGCCGGCCT
XylR	TTCACTAAGAGACACCGCATCACCCTTCTGTTCAATGCCAACAAAGCATACGATCGACAGGTCGTAGAGGGAGT<br>GGGGGAATACCTTCAGGCCTCTCAGAGCGAGTGGGACATTTTCATAGAAGAAGATTTTAGAGCTCGTATAGATA<br>AGATCAAGGACTGGCTAGGCGACGGGGTAATTGCTGACTTTGATGACAAACAGATCGAGCAGGCCTTGGCTGAT<br>GTGGACGTCCCCATTGTGGGCGTAGGCGGCTCCTATCACTTAGCCGAGTCCTACCCACCAGTCCATTATATAGCC<br>ACGGACAACTACGCCTTGGTGGAGAGTGCTTTCCTGCACCTGAAAGAGAAGGGCGTGAATAGATTTGCTTTCTAT<br>GGGCTGCCCGAAAGTTCGGGTAAACGCTGGGCGACTGAGCGGGAGTACGCATTTAGACAGTTAGTTGCCGAGGA<br>GAAATACAGAGGAGTGGTCTACCAGGGTTTGGAAACCGCACCTGAAAATTGGCAGCACGCACAGAACAGGCTG<br>GCCGATTGGCTGCAGACCCTTCCCCCTCAGACAGGAATTATAGCTGTTACAGACGCCAGGGCCAGGCATATCCTG<br>CAGGTTTGCGAGCACTTACACATCCCGGTGCCAGAAAAACTCTGTGTCATCGGCATTGATAACGAAGAACTCACG

	AGGTACCTGAGCCGCGTGGCACTGTCCTCCGTTGCCCAAGGTGCCAGACAAATGGGATATCAAGCGGCCAAGCT GCTCCATAGGCTGCTCGACAAAGAAGAAATGCCTCTTCAGCGGATTCTCGTTCCCCCAGTCCGGGTAATCGAGCG CCGATCTACTGATTATAGGAGCCTCACAGACCCTGCTGTTATCCAAGCAATGCATTATATCCGTAATCACGCATG TAAGGGGATTAAGGTGGACCAGGTGCTGGATGCGGTGGGGATTTCTCGAAGTAATTTGGAGAAGCGGTTTAAGG AGGAGGTGGGCGAGACAATCCACGCTATGATTCATGCTGAGAAACTGGAGAAGGCCCGGTCTCTTCTCATCTCA ACCACCCTGAGCATAAACGAAATCAGCCAAATGTGCGGATACCCCTCATTGCAGTATTTCTACTCCGTCTTTAAG AAGGCATACGATACCACTCCGAAAGAATATCGCGACGTGAACTCAGAGGTGATGCTC

Table S4 | Plasmids and details for transient transfection experiments

Indicated is the amount of plasmid transfected per well of a 96-well plate.

Figure 1b	75 ng of plasmid coding for constitutively expressed transcription factor (pOM217, pOM218, pOM219, pOM235, pOM236, pOM237) and 75 ng of reporter plasmid (pOM213)
Figure 1d	75 ng of plasmid coding for constitutively expressed transcription factor (pOM219) and 75 ng of reporter plasmid (pOM211, pOM12, pOM213, pSG164, pSG165, pSG166, pSG167)
Figure 2b	75 ng of plasmid coding for constitutively expressed transcription factor (pOM219) and 75 ng of reporter plasmid (pOM213, pOM247, pOM248, pOM249, pOM250)
Figure 2c	75 ng of plasmid coding for constitutively expressed transcription factor (pOM219) and 75 ng of reporter plasmid (pOM250, pSG089, pSG098)
Figure 2d	75 ng of plasmid coding for constitutively expressed transcription factor (pOM219) and 75 ng of reporter plasmid (pSG098)
Figure 3b	50 ng of plasmid coding for constitutively expressed transcription factor (pOM219), 50 ng of reporter plasmid (pSG098), 50 ng

	of constitutively expressed xylose transporter (pSG126) or empty plasmid
Figure 3c	230 ng of plasmid coding for constitutively expressed transcription factor (pOM219), 230 ng of reporter plasmid (pSG098), 230 ng of constitutively expressed xylose transporter (pSG126). The experiment was carried out in a 24-well plate format.
Figure 3d	230 ng of plasmid coding for constitutively expressed transcription factor (pOM219), 230 ng of reporter plasmid (pSG098), 230 ng of constitutively expressed xylose transporter (pSG126). The experiment was carried out in a 24-well plate format.
Figure 3e	50 ng of plasmid coding for constitutively expressed transcription factor (pOM219), 50 ng of reporter plasmid (pSG098), 50 ng of constitutively expressed xylose transporter (pSG126) or empty plasmid (-ctrl)
Figure 4b	10 µg of plasmid coding for constitutively expressed transcription factor (pOM219), 10 µg of reporter plasmid (pSG098), 10 µg of constitutively expressed xylose transporter (pSG126). Cells were transfected in 150 mm culture dish.
Figure 4c	10 µg of plasmid coding for constitutively expressed transcription factor (pOM219), 10 µg of reporter plasmid (pSG098), 10 µg of constitutively expressed xylose transporter (pSG126). Cells were transfected in 150 mm culture dish.
Figure S1	75 ng of plasmid coding for constitutively expressed transcription factor (pOM217, pOM218, pOM219, pOM235, pOM236, pOM237) and 75 ng of reporter plasmid (pSG098)
Figure S2	50 ng of plasmid coding for constitutively expressed transcription factor (pOM219), 50 ng of reporter plasmid (pSG098), 50 ng of constitutively expressed xylose transporter (pSG125) or empty plasmid
Figure S4	15 µg of plasmid coding for SWEET (pSG156) and 15 µg of empty plasmid. Cells were transfected in 150 mm culture dish.
Figure S6	75 ng of plasmid coding for SWEET (pSG151) and 75 ng of empty plasmid, or 50 ng of pOM219, pSG098, pSG126
Figure S9	Insulin-producing cells: 75 ng of plasmid coding for $SWEET_{ins}$ (pSG156) and 75 ng of empty plasmid. insulin assay: 50 ng of pIR, pLeo665, MKp37 or pLeo665, MKp37, empty plasmid.

Supplementary References

Haellman, V., Strittmatter, T., Bertschi, A., Stücheli, P. & Fussenegger, M. A versatile plasmid architecture for mammalian synthetic biology (VAMSyB). *Metab. Eng*. **66**, 41– 50 (2021).

Jacob, K. K., Whittaker, J. & Stanley, F. M. Insulin receptor tyrosine kinase activity and phosphorylation of tyrosines 1162 and 1163 are required for insulin-increased prolactin gene expression. *Mol. Cell. Endocrinol*. **186**, 7–16 (2002).

Keeley, M. B., Busch, J., Singh, R. & Abel, T. TetR hybrid transcription factors report cell signaling and are inhibited by doxycycline. *Biotechniques* **39**, 529–535 (2005).

Scheller, L., Strittmatter, T., Fuchs, D., Bojar, D. & Fussenegger, M. Generalized extracellular molecule sensor platform for programming cellular behavior article. *Nat. Chem. Biol*. **14**, 723–729 (2018).
